# Nortriptyline Inhibits Lysosomal Exocytosis‐Mediated SASP During Gastric Cancer Progression via Targeting HOXA1‐PITX2 Phase Separation

**DOI:** 10.1002/advs.202512407

**Published:** 2025-09-25

**Authors:** Yi Zhou, Chunhui Yang, Xinyue Li, Xiaojing Wang, Wanju Jiao, Xiaolin Wang, Jiaying Qu, Bosen Zhao, Shunchen Zhou, Qiangsong Tong, Liduan Zheng

**Affiliations:** ^1^ Department of Pathology, Union Hospital, Tongji Medical College Huazhong University of Science and Technology 1277 Jiefang Avenue Wuhan Hubei Province 430022 P. R. China; ^2^ Department of Pediatric Surgery, Union Hospital, Tongji Medical College Huazhong University of Science and Technology 1277 Jiefang Avenue Wuhan Hubei Province 430022 P. R. China; ^3^ Department of Geriatrics, Union Hospital, Tongji Medical College Huazhong University of Science and Technology 1277 Jiefang Avenue Wuhan Hubei Province 430022 P. R. China

**Keywords:** cancer progression, homeobox A1, lysosomal exocytosis, paired like homeodomain 2, senescence‐ associated secretory phenotype

## Abstract

Lysosomal exocytosis, a calcium‐dependent secretory process, facilitates extracellular release of cargos that promote cancer progression, though its regulatory pathways and therapeutic strategies are poorly understood. Herein, using combined transcriptomic and proteomic approaches, we identify homeobox A1 (HOXA1) as a functional partner of paired like homeodomain 2 (PITX2) within biomolecular condensates forming via liquid–liquid phase separation. Mechanistically, HOXA1‐PITX2 complex facilitates the expression of mucolipin 1 *(MCOLN1*) and RAS‐related protein Rab‐3A (*RAB3A*), which drive lysosomal exocytosis of galectin‐1 (LGALS1) and insulin like growth factor binding protein 7 (IGFBP7) from senescent gastric cancer cells. This process potentiates AKT activation and epithelial‐mesenchymal transition, accelerating tumorigenesis and aggressiveness of gastric cancer. Molecular docking and affinity purification assays reveal nortriptyline (Nor) as a potent phase separation disruptor of HOXA1‐PITX2 complex. Preclinical studies demonstrate that Nor administration attenuates lysosomal exocytosis‐mediated senescence‐associated secretory phenotype (SASP) and reduces aggressive phenotypes in gastric cancer models, underscoring the *HOXA1*/*PITX2* axis as a critical regulator of gastric cancer progression. Clinically, elevated expression of *HOXA1*, *PITX2*, *MCOLN1*, *RAB3A*, *LGALS1*, and *IGFBP7* constitutes a prognostic signature correlating with poor outcomes of gastric cancer patients. Collectively, these results indicate that Nor impedes gastric cancer progression by suppressing HOXA1‐PITX2 phase separation and subsequent lysosomal exocytosis‐mediated SASP.

## Graphical abstract

This study identifies that HOXA1 facilitates transcriptional activity of PITX2 in liquid condensates, resulting in up‐regulation of *MCOLN1* and *RAB3A* followed by lysosomal exocytosis of LGALS1 and IGFBP7 from senescent cells, thus activating AKT signaling and EMT. ‐Blocking HOXA1‐PITX2 interaction by nortriptyline suppresses progression of gastric cancer, highlighting *HOXA1*/*PITX2* axis as a novel therapeutic target for lysosomal exocytosis‐mediated SASP in cancers.

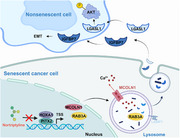

## 1 Introduction

Gastric cancer is a highly aggressive malignancy and ranks as the second leading cause of global cancer‐related death.^[^ ^1^ ^]^ Although its mortality has declined over the past 50 years as a result of successful treatment of *H. pylori* infection, five‐year survival rates of gastric cancer patients in advanced stages persist at discouragingly low levels, largely owing to recurrent or metastatic events,^[^ ^1^, ^2^ ^]^ indicating the urgency for exploring the underlying mechanisms and therapeutic approach. Previous proteomic studies have identified a series of soluble senescence‐associated secretory phenotype (SASP) proteins from human cells, including cytokines, growth factors, insulin like growth factor binding proteins (IGFBPs), C‐X‐C motif chemokine ligands, matrix metalloproteinases (MMPs), and tissue inhibitors of metallopeptidase (TIMPs).^[^ ^3^ ^]^ Among them, IGFBP5 is up‐regulated in senescent cells and exerts paracrine pro‐senescence effects on healthy mesenchymal stromal cells (MSCs) via interaction with retinoic acid receptors.^[^ ^4^ ^]^ As a central SASP component, IGFBP7 propagates senescence of MSCs via modulating insulin, insulin like growth factor, or activin A signaling pathways.^[^ ^5^ ^]^ Emerging evidences show that SASP exerts potent pro‐tumorigenic or tumor suppressive functions in a context‐dependent manner.^[^ ^6^ ^]^ For example, SASP factors interleukin 6 (IL‐6) and interleukin 8 facilitate epithelial‐mesenchymal transition (EMT) and invasive potential of pre‐malignant cells.^[^ ^7^ ^]^ Inflammation‐driven SASP in cancer‐associated fibroblasts enhances the peritoneal dissemination of gastric cancer.^[^ ^8^ ^]^ Meanwhile, RAS‐induced senescent hepatocytes facilitate recruitment of myeloid cells via secreting C‐C motif chemokine ligand 2, resulting in repression of cancer development.^[^ ^9^ ^]^ However, the regulatory mechanisms and targeting strategies of SASP during advancement of gastric malignancies warrant comprehensive elucidation.

Lysosomal exocytosis, a calcium‐regulated and multifaceted process, involves the fusion of lysosomes with plasma membrane (PM), leading to extracellular release of enzymes or metabolites.^[^ ^10^ ^]^ Once lysosomes transport from perinuclear region to cellular periphery along microtubules, the exocytosis of luminal contents is triggered by mucolipin 1 (*MCOLN1*)‐mediated calcium ion (Ca^2^⁺) release, which mediates PM repair, extracellular matrix reorganization, or phagocytic clearance.^[^ ^10^ ^]^ In addition, lysosomal exocytosis depends on lysosomal‐associated membrane protein 1 (LAMP1), as its knockdown inhibits the docking of lysosome to PM.^[^ ^11^ ^]^ Of note, cancer cells exploit dysregulated lysosomal exocytosis of hydrolases (such as cathepsins) or chemotherapeutic agents to gain survival advantage, matrix invasion, pro‐metastatic dissemination, or multidrug resistance.^[^ ^10^ ^]^ In pancreatic ductal adenocarcinoma, inositol polyphosphate‐4‐phosphatase type II B over‐expression drives the generation of phosphatidylinositol 3,5‐bisphosphate on lysosomes to facilitate *MCOLN1*‐mediated Ca^2^⁺ release and lysosomal exocytosis, which is hijacked to enhance cellular motility and invasiveness.^[^ ^12^ ^]^ Transmembrane protein 106B modulates the expression of lysosomal genes, and promotes lysosomal exocytosis of cathepsins necessary for lung cancer progression.^[^ ^13^ ^]^ These studies indicate that dysregulation of lysosomal exocytosis represents an emergent therapeutic vulnerability of cancers.

In current investigation, we identify paired like homeodomain 2 (PITX2) as an essential transcriptional coordinator of lysosomal exocytosis genes *MCOLN1* and RAS‐related protein Rab‐3A (*RAB3A*), which is linked to an adverse outcome in gastric cancer. Notably, PITX2 facilitates the SASP in serum deprivation‐induced senescent cells, leading to an increase in intracellular free calcium ions followed by anterograde movement and PM fusion of lysosomes, which promotes lysosomal exocytosis, aggressive proliferation, migration, and dissemination of gastric cancer cells. Notably, serum deprivation triggers direct interaction of homeobox A1 (HOXA1) with PITX2 in liquid condensates, resulting in increased PITX2 activity and lysosomal exocytosis. Pre‐clinically, administration of nortriptyline (Nor) blocks the interaction of HOXA1 with PITX2, and suppresses the lysosomal exocytosis, tumor formation, and aggressive behavior, underscoring the functions of *HOXA1*/*PITX2* axis in lysosomal exocytosis‐mediated SASP and cancer progression.

## 2 Results

### 2.1 *PITX2* Facilitates Gene Expression Essential for Lysosomal Exocytosis in Gastric Cancer

**Figure 1:**
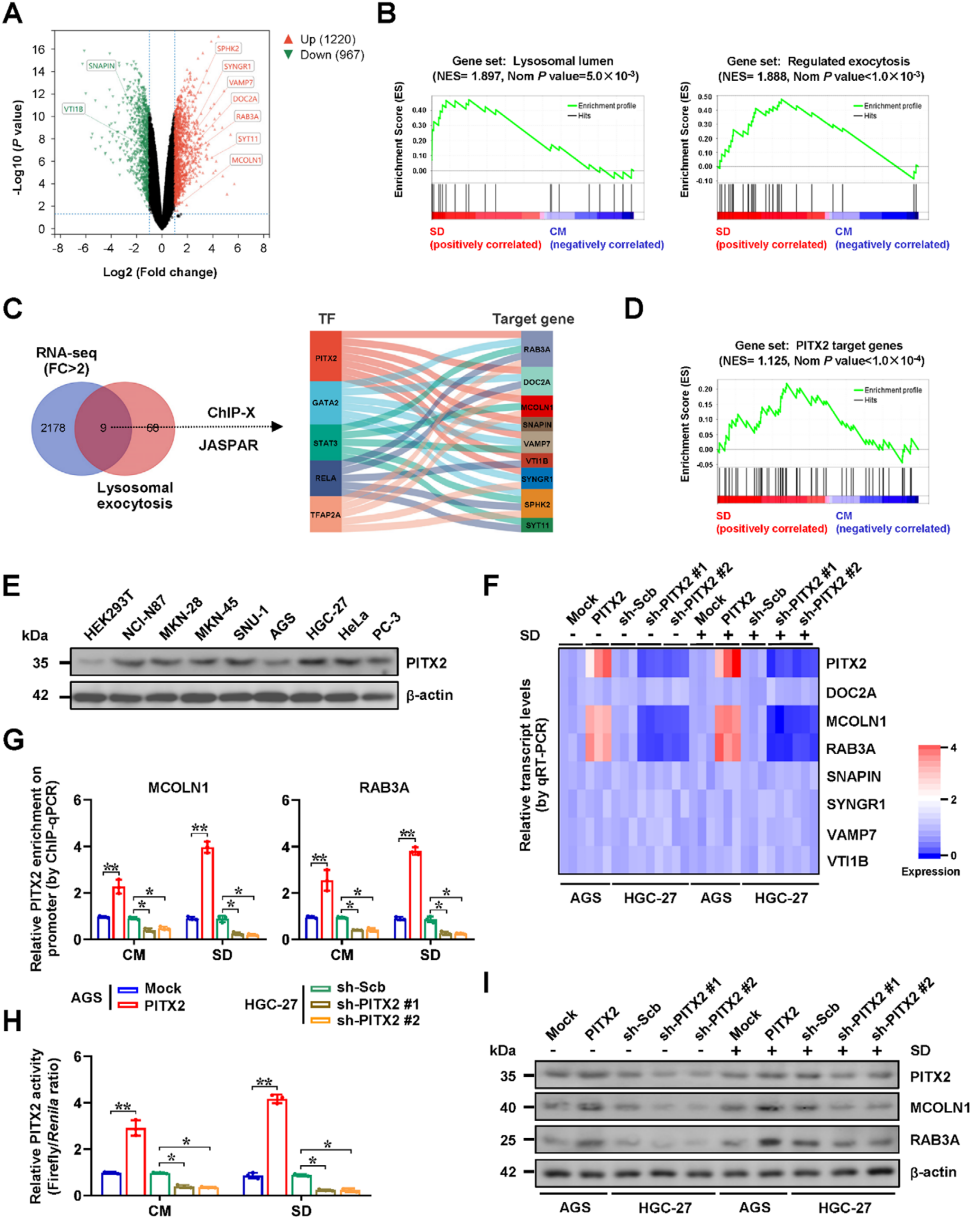
*PITX2* facilitates gene expression essential for lysosomal exocytosis in gastric cancer. A) Volcano plot of RNA‐seq assay indicating the differentially expressed genes (fold change >2, *p *<0.05) in gastric cancer AGS cells treated with complete medium (CM) or serum deprivation (SD) for 10 days (*n *= 3). B) GSEA revealing the involvement of differentially expressed genes in regulation of lysosomal lumen or exocytosis. C) Venn diagram (left panel) and Sankey diagram (right panel) showing the identification of differentially expressed lysosomal exocytosis genes and their potential transcription factors (TF) analyzed by ChIP‐X and JASPAR (http://jaspar.genereg.net/) programs. D) GSEA indicating PITX2 targets in differentially expressed genes of gastric cancer AGS cells treated with CM or SD for 10 days. E) Western blot assay showing the PITX2 levels in cultured HEK293T cells and cancer cell lines. F) Heatmap of real‐time qRT‐PCR assay indicating the levels (normalized to *β‐actin*, *n *= 5) of *DOC2A*, *MCOLLN1*, *RAB3A*, *SNAPIN*, *SYNGR1*, *VAMP7*, and *VI1B* in AGS and HGC‐27 cells stably transfected with empty vector (mock), *PITX2*, scramble shRNA (sh‐Scb), sh‐PITX2 #1, or sh‐PITX2 #2 under CM or SD condition. G‐I) ChIP‐qPCR assay (G, normalized to input, *n *= 3), dual‐luciferase (H, *n *= 3), and western blot (I) assays showing the PITX2 enrichment on *MCOLLN1* or *RAB3A* promoter region, PITX2 activity, and protein levels of MCOLLN1 and RAB3A in AGS and HGC‐27 cells stably transfected with mock, *PITX2*, sh‐Scb, sh‐PITX2 #1, or sh‐PITX2 #2 under CM or SD condition. Fisher's exact test for overlapping analysis in C. Student's t‐test or one‐way ANOVA compared the difference in G and H. Data are shown as mean ± s.e.m. (error bars); ^*^, *p *<0.05; ^**^, *p *<0.01.

To explore the critical regulators of cancer progression under stress conditions, transcriptome profiling analysis was conducted and identified 1220 elevated and 967 down‐regulated genes (fold change >2, *p *<0.05) in gastric cancer AGS cells subjected to serum deprivation (SD, **Figure** **1**A). Gene set enrichment analysis (GSEA) demonstrated that these genes exhibited significant enrichment in pathways related to lysosomal lumen [normalized enrichment score (NES) = 1.897, normalized *P* = 5.0 × 10^−3^] or exocytosis (NES = 1.888, normalized *p *<1.0 × 10^−3^, Figure 1B). A comprehensive analysis using the ChIP‐X program,^[^ ^14^ ^]^ JASPAR (http://jaspar.genereg.net/) program, and GSEA further identified PITX2 as the top transcriptional regulator of lysosomal exocytosis based on the number of target genes (Figure 1C,D; and Table S1, Supporting Information). Analysis of public datasets from Kaplan‐Meier Plotter database (http://kmplot.com/) indicated that gastric cancer individuals exhibiting elevated *PITX2* expression had reduced overall (*P *= 1.5 × 10^−3^) or event‐free (*P *= 9.0 × 10^−4^) survival rates (Figure S1A, Supporting Information). Elevated *PITX2* expression was found in established gastric cancer cell lines (Figure 1E; Figure S1B, Supporting Information). To examine the role of *PITX2* in regulating gene expression essential for lysosomal exocytosis, AGS, MKN‐45 (relatively low *PITX2* expression), HGC‐27, and SNU‐1 (relatively high *PITX2* expression) cells were selected as models. Notably, steady ectopic expression or silencing of *PITX2* led to elevated or reduced transcript levels of *MCOLN1* or *RAB3A*, but not of double C2 domain alpha (*DOC2A*), SNAP associated protein (*SNAPIN*), synaptogyrin 1 (*SYNGR1*), vesicle associated membrane protein 7 (*VAMP7*), or vesicle transport through interaction with t‐SNAREs 1B (*VTI1B*), in AGS and HGC‐27 cells under complete medium (CM) or SD condition (Figure 1F). Chromatin immunoprecipitation (ChIP)‐qPCR, dual‐luciferase reporter, and western blot analyses demonstrated that PITX2 enrichment on *MCOLN1* or *RAB3A* promoter region, its transcriptional activity, and protein levels of MCOLN1 or RAB3A were elevated or reduced in AGS, MKN‐45, HGC‐27, or SNU‐1 cells with steady transfection of either *PITX2* or short hairpin RNA (shRNA) against *PITX2* (sh‐PITX2) under CM or SD condition (Figure 1G–I; Figure S1C–E, Supporting Information). Furthermore, under CM or SD condition, enforced over‐expression or silencing of *PITX2* led to decrease or increase in senescence‐associated β‐gal (SA‐β‐gal)^+^/EdU^−^ staining rates and heterochromatin protein 1 gamma (HP1γ)‐positive heterochromatic lesions in AGS and HGC‐27 cells, respectively (Figure S2A, Supporting Information). Ectopic expression of *PITX2* prevented the up‐regulation of senescent markers (P16 and P21)^[^ ^15^ ^]^ in AGS cells treated with prolonged SD (Figure S2B, Supporting Information). These findings suggested that PITX2, functioning as a transcription factor, increased the levels of lysosomal exocytosis‐related genes in gastric cancer.

### 2.2 *PITX2* Promotes Lysosomal Exocytosis of Senescent Gastric Cancer Cells Via Up‐Regulating *MCOLN1* and *RAB3A*

**Figure 2:**
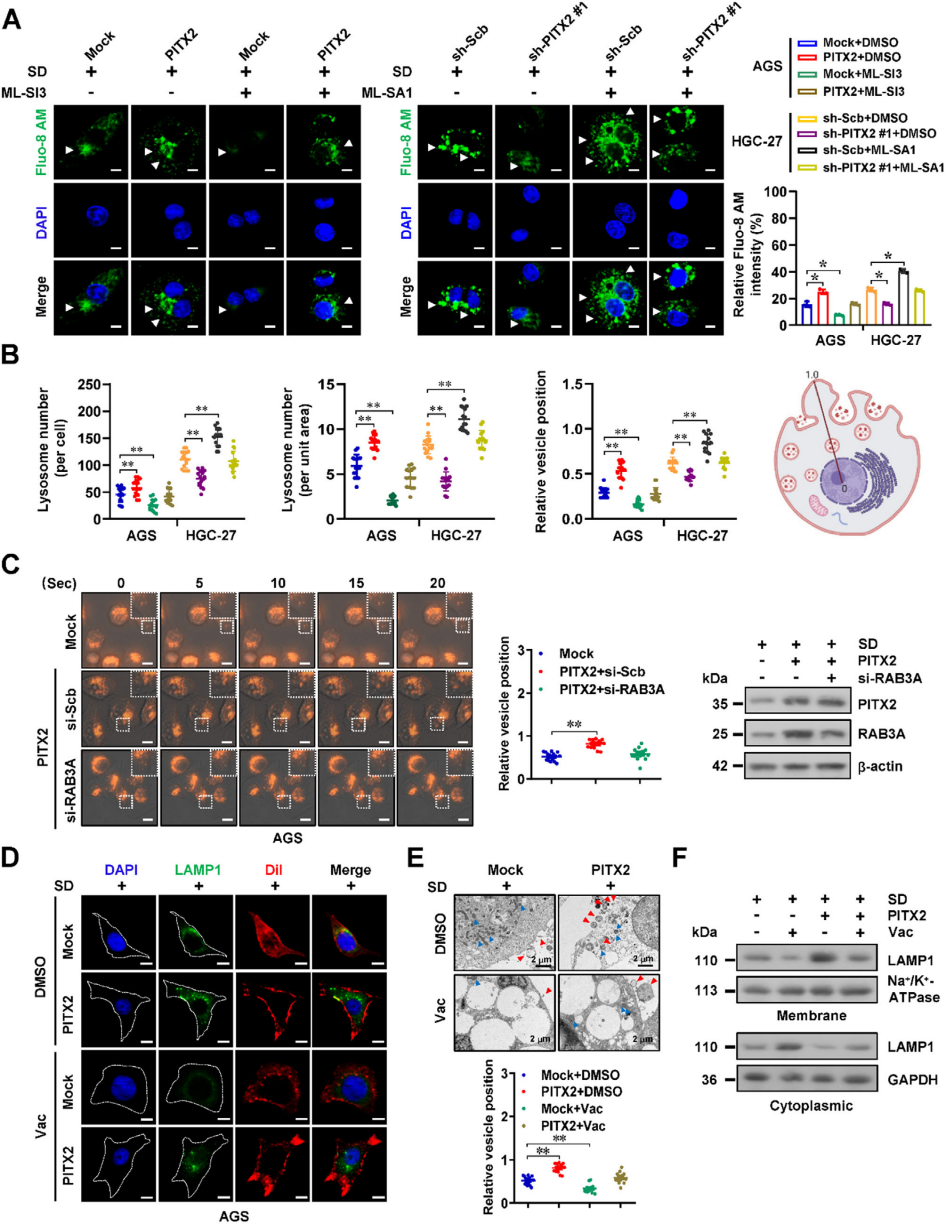
*PITX2* promotes lysosomal exocytosis of senescent gastric cancer cells via up‐regulating *MCOLN1* and *RAB3A*. A) Representative images (left panel) and quantification (right panel) of Fluo 8‐AM staining (arrowheads) in AGS and HGC‐27 cells stably transfected with empty vector (mock), *PITX2*, scramble shRNA (sh‐Scb), sh‐PITX2 #1, or sh‐PITX2 #2 under SD condition, and those treated with ML‐SI3 (10 µmol·L^−1^) or ML‐SA1 (20 µmol·L^−1^), with nuclei staining by DAPI. Scale bars: 10 µm. B) Quantification of Lyso‐Tracker Red staining (left panel) showing the number and anterograde transport distance of lysosomes in AGS and HGC‐27 cells stably transfected with mock, *PITX2*, sh‐Scb, sh‐PITX2 #1, or sh‐PITX2 #2 under SD condition (*n *= 15), and those treated with ML‐SI3 (10 µmol·L^−1^) or ML‐SA1 (20 µmol·L^−1^). Schematic diagram (right panel) for calculating the distance of lysosome to plasma membrane. C) Confocal images and quantification of Lyso‐Tracker Red staining (left panel, *n *= 15) and western blot (right panel) assays indicating the lysosomal anterograde transport and expression levels of PITX2 or RAB3A in living AGS cells stably transfected with mock or *PITX2*, and those co‐transfected with scramble siRNA (si‐Scb) or si‐RAB3A. Scale bars: 10 µm. D) Representative images of LAMP1 and Dil staining in AGS cells stably transfected with mock or *PITX2* under SD condition, and those treated with vacuolin‐1 (Vac, 1.0 µmol·L^−1^). Scale bars: 10 µm. E) Transmission electron microscopic observation (upper panel) and quantification (lower panel, *n *= 15) of lysosomal vesicles (red arrowheads) in AGS cells stably transfected with mock or *PITX2* under SD condition, and those treated with Vac (1.0 µmol·L^−1^). Scale bars: 2 µm. F) Western blot assay indicating the plasma membrane or cytoplasmic expression of LAMP1 in AGS cells stably transfected with mock or *PITX2* under SD condition, and treated with Vac (1.0 µmol·L^−1^). One‐way ANOVA compared the difference in A‐C and E. Data are shown as mean ± s.e.m. (error bars); ^*^, *p *<0.05; ^**^, *p *<0.01.

Since *MCOLN1* and *RAB3A* contribute to Ca^2+^ release and trafficking essential for lysosomal exocytosis,^[^ ^16^ ^]^ we further determined whether *PITX2* could impact the biological characteristics of senescent gastric cancer cells. Under SD condition, sustained over‐expression or silencing of *PITX2* significantly enhanced or reduced intracellular calcium concentrations and lysosome count or anterograde transport distance, which were reversed by mucolipin‐specific synthetic inhibitor 3 (ML‐SI3) or agonist 1 (ML‐SA1), respectively (**Figures** **2**A,B, and S2C, Supporting Information). Consistent with the roles of *MCOLN1* in autophagy regulation,^[^ ^17^ ^]^ there was significant decrease or increase of autophagic flux, evidenced by reduction or accumulation of autolysosomes, along with increased or decreased levels of autophagosomes, microtubule associated protein 1 light chain 3 (LC3) I/II, and sequestosome 1 (SQSTM1), in AGS or HGC‐27 cells with stable over‐expression or silencing of *PITX2* under SD condition, which was abolished by ML‐SI3 or ML‐SA1 treatment (Figure S3A–C, Supporting Information). Meanwhile, knockdown of autophagy related 5 (*ATG5*), a protein contributing to autophagosome formation,^[^ ^18^ ^]^ led to increase in lysosomal anterograde transport distance and LAMP1 expression at the PM in AGS cells with SD treatment (Figure S3D–F, Supporting Information). In Lyso‐Tracker Red staining assay, *RAB3A* knockdown also abolished the enhancement of lysosomal anterograde transport in AGS cells with *PITX2* over‐expression (Figure 2C). Additionally, steady over‐expression or silencing of *PITX2* facilitated or inhibited the LAMP1 localization near PM in AGS and HGC‐27 cells under SD condition. This effect was attenuated by the lysosomal exocytosis inhibitor (vacuolin‐1) or activator (ionomycin, Figure 2D; Figure S4A, Supporting Information). Transmission electron microscopy revealed an increased number of lysosomes near the PM in AGS cells stably transfected with *PITX2*, an effect reversed by vacuolin‐1 (Figure 2E). Notably, consistent with previous studies,^[^ ^19^ ^]^ vacuolin‐1 treatment induced vacuole formation without altering intracellular calcium levels (Figure 2E; Figure S4B, Supporting Information). Western blot analysis further demonstrated that stable ectopic expression or silencing of *PITX2* led to elevation or reduction of LAMP1 expression at the PM in AGS and HGC‐27 cells, with these effects abolished by vacuolin‐1 or ionomycin (Figure 2F; Figure S4C, Supporting Information). Mining the Kaplan‐Meier Plotter database revealed that up‐regulation of *MCOLN1* (*P *= 4.6 × 10^−2^ and 4.4 × 10^−2^), *RAB3A* (*P *= 7.4 × 10^−7^ and 5.5 × 10^−6^), or *LAMP1* (*P *= 3.2 × 10^−3^ and 2.3 × 10^−2^) was associated with poor overall or event‐free survival in gastric cancer patients (Figure S4D, Supporting Information). These findings indicated that *PITX2* enhanced lysosomal exocytosis in senescent gastric cancer cells via up‐regulating *MCOLN1* and *RAB3A*.

### 2.3 *PITX2* Drives Tumorigenesis and Aggressiveness of Gastric Cancer Via Lysosomal Exocytosis‐Mediated SASP

**Figure 3:**
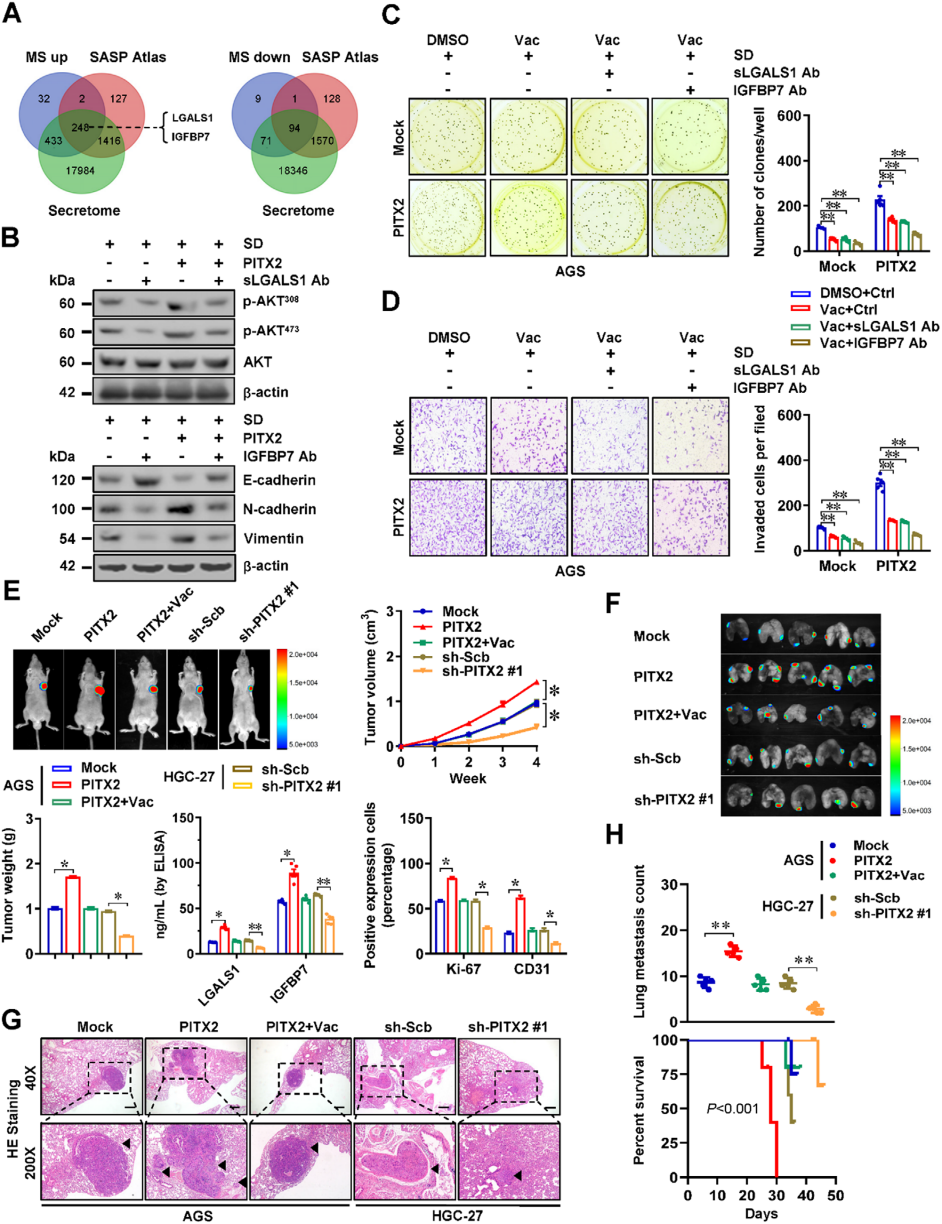
*PITX2* drives tumorigenesis and aggressiveness of gastric cancer via lysosomal exocytosis‐mediated SASP. A) Venn diagram showing the over‐lapping analysis of altered proteins in mass spectrometry (MS) analysis of culture medium from AGS cells with stable *PITX2* over‐expression or vacuoloin‐1 (1.0 µmol·L^−1^) treatment under SD condition, SASP proteins in SASP Atlas database (http://www.SASPAtlas.com), and reported secretome. B) Western blot assay indicating the levels of p‐AKT^308^, p‐AKT^473^, E‐cadherin, N‐cadherin, or Vimentin in AGS cells with stably transfected with empty vector (mock) or *PITX2* under SD condition, and those treated with neutralizing antibody against sLGALS1 or IGFBP7. C and D) Representative images (left panel) and quantitative (right panel) of soft agar (C) and matrigel invasion (D) assays showing the growth and invasion of AGS cells treated with culture medium from those stably transfected with mock or *PITX2* under SD condition, and treated with vacuoloin‐1 (Vac, 1.0 µmol·L^−1^) and neutralizing antibody against sLGALS1 or IGFBP7 (*n *= 5). E) Representative images (left upper panel), growth curve (right upper panel), weight at the end points (lower panel), LGALS1 and IGFBP7 secretion (lower panel), and Ki‐67 or CD31 expression (lower panel) of xenograft tumors formed by subcutaneous injection of AGS or HGC‐27 cells in nude mice (*n *= 5 for each group) that subsequently treated with intravenous injection of culture medium collected from senescent cells stably transfected with mock, *PITX2*, scramble shRNA (sh‐Scb), or sh‐PITX2 #1, with or without Vac (1.0 µmol·L^−1^) treatment. F‐H) Representative images (F), hematoxylin‐eosin (HE) staining (G, arrowheads), quantification of lung metastatic colonization (H), and Kaplan‐Meier curves (H) of nude mice (*n *= 5 for each group) receiving vein tail injection of AGS or HGC‐27 cells that subsequently treated with intravenous injection of culture medium collected from senescent cells stably transfected with mock, *PITX2*, sh‐Scb, sh‐PITX2 #1, with or without Vac (1.0 µmol·L^−1^) treatment. One‐way ANOVA or Student's t‐test compared the difference in C‐E and H. Log‐rank test for survival comparison in H. Data are shown as mean ± s.e.m. (error bars); ^*^, *p *<0.05; ^**^, *p *<0.01.

Subsequently, we explored the essential protein composition associated with *PITX2*‐induced lysosomal exocytosis via quantitative proteomic analysis. In response to SD treatment, mass spectrometry identified 715 up‐regulated and 175 down‐regulated proteins in the culture medium of AGS cells with stable *PITX2* over‐expression, which were reduced or enhanced by vacuolin‐1 treatment, respectively (Figure S5A, Supporting Information). A comprehensive overlap analysis with SASP proteins from SASP Atlas database (http://www.SASPAtlas.com)^[^ ^3^ ^]^ and previously reported secretomes^[^ ^20^ ^]^ identified 248 up‐regulated and 94 down‐regulated candidates involved in phosphoinositide 3‐kinase (PI3K)/protein kinase B (AKT) signaling, vesicle‐mediated transport, or IGFBP signaling (**Figure** **3**A; Figure S5B and Tables S2 and S3, Supporting Information), including galectin‐1 (LGALS1) and IGFBP7 (Figure S5C, Supporting Information). Notably, elevated levels of *LGALS1* and *IGFBP7* were linked to an adverse survival prognosis in individuals with gastric cancer from Kaplan‐Meier Plotter database (Figure S5D, Supporting Information). Enzyme‐linked immunosorbent assay (ELISA) confirmed the increase of LGALS1 and IGFBP7 levels in the culture medium of AGS cells stably transfected with *PITX2* under SD condition, which was abolished by vacuolin‐1 treatment (Figure S6A, Supporting Information). Consistent with the roles of LGALS1 and IGFBP7 in promoting AKT phosphorylation^[^ ^21^ ^]^ and EMT,^[^ ^22^ ^]^ treatment with culture medium from senescent AGS cells stably over‐expressing *PITX2* led to increased p‐AKT^308^ and p‐AKT^473^ levels, as well as up‐regulation of mesenchymal markers (N‐cadherin or Vimentin), alongside a decrease in epithelial marker (E‐cadherin) in AGS cells (Figure 3B; Figure S6B, Supporting Information). These effects were attenuated by vacuolin‐1 treatment and neutralizing antibodies against LGALS1 or IGFBP7 (Figure 3B; Figure S6B, Supporting Information). Furthermore, incubation with culture medium from senescent cells stably over‐expressing *PITX2* enhanced the proliferative and invasive potential of AGS cells, which was negated upon treatment with vacuolin‐1 and neutralizing antibodies against LGALS1 or IGFBP7 (Figure 3C,D). In nude mice, administration of culture medium derived from senescent AGS cells stably over‐expressing *PITX2* significantly promoted the growth, weight, LGALS1 and IGFBP7 secretion, Ki‐67‐based proliferation index and CD31‐positive microvasculature in AGS‐derived xenograft tumors, accompanied by alterations in levels of AKT phosphorylation and EMT markers, which were reversed by vacuolin‐1 injection (Figure 3E and S6C,D). In experimental metastasis assays, administration of culture medium collected from senescent cells with stable *PITX2* over‐expression via tail vein facilitated lung metastasis of AGS cells in athymic nude mice, resulting in reduced survival rates, which was attenuated by vacuolin‐1 treatment (Figure 3F–H). In contrast, administration of culture medium from senescent HGC‐27 cells with stable *PITX2* silencing suppressed tumor formation and aggressiveness of cancer cells in vivo (Figure 3E–H; Figure S6C,D, Supporting Information). Collectively, these findings suggested that *PITX2* promoted tumorigenesis and aggressiveness of gastric cancer via lysosomal exocytosis‐mediated SASP.

### 2.4 HOXA1 Interacts with PITX2 to Facilitate Lysosomal Exocytosis‐Related Gene Expression in Gastric Cancer Cells

**Figure 4:**
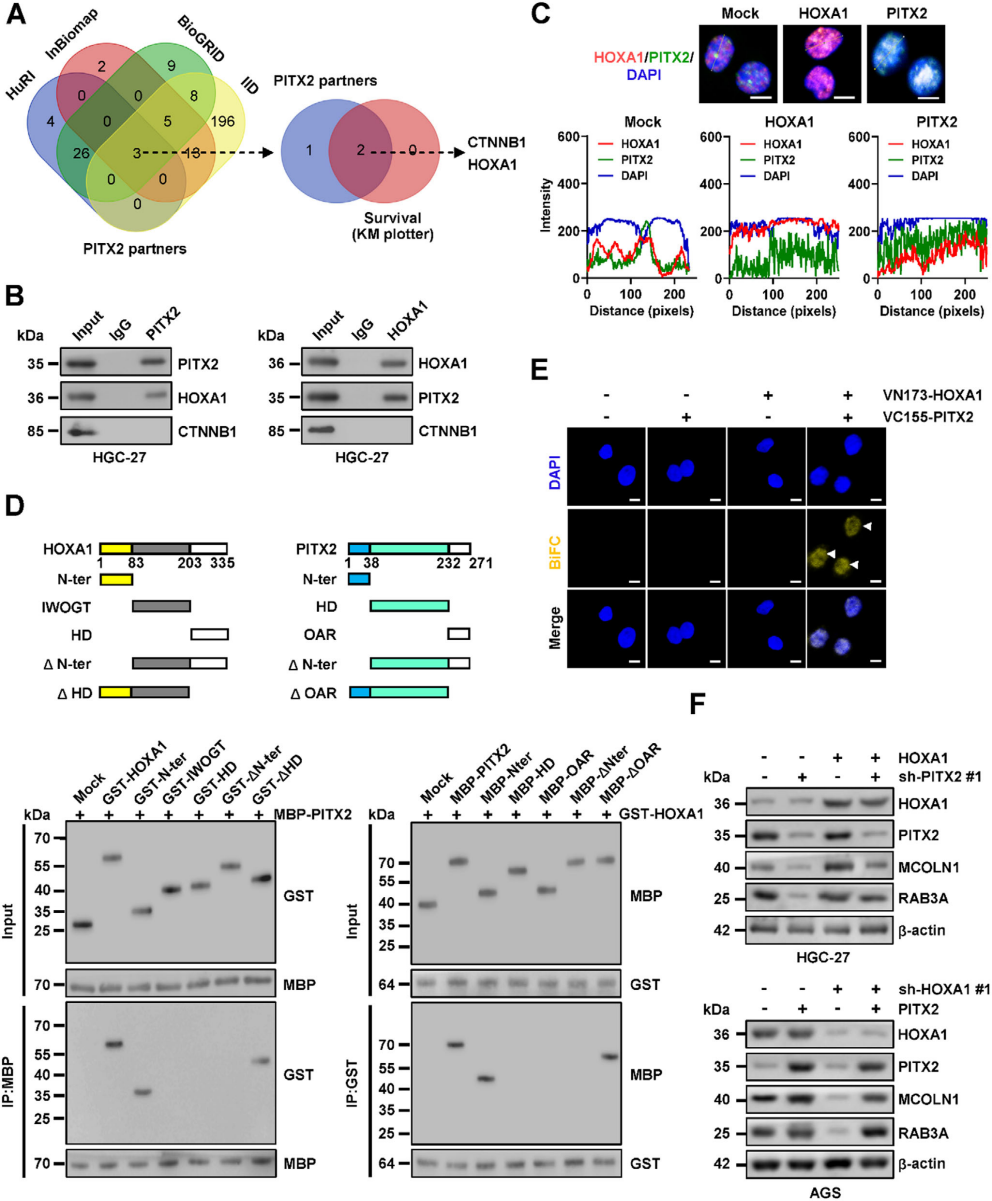
HOXA1 interacts with PITX2 protein to facilitate lysosomal exocytosis‐related gene expression in gastric cancer cells. A) Venn diagram showing the identification of consistent PIXT2 partners via comprehensive analysis of public databases HuRI (http://www.interactome‐ atlas.org/), InBioMap (https://inbio‐discover.com/), BioGRID (https://thebiogrid.org/), and IID (http://iid.ophid.utoronto.ca/). B) Co‐IP and Western blot analysis revealed endogenous interactions between PITX2 and HOXA1 proteins in HGC‐27 cells. IgG binding protein was used as a negative control. C) Representative images (upper panel) and quantification (lower panel) of immunofluorescence showing co‐localization of PITX2 and HOXA1 in AGS cells, as well as in those stably transfected with empty vectors (mock), *HOXA1*, or *PITX*2. Scale bars: 10 µm. D) Co‐IP and Western blot assays indicating interaction between GST‐tagged HOXA1 and MBP‐tagged PITX2 truncation proteins as indicated. E) Representative images of BiFC assay showing physical interaction (arrowheads) of PITX2 and HOXA1 in AGS cells co‐transfected with VN173‐HOXA1 and VC155‐PITX2. Scale bars: 10 µm. F) Western blot assay showing the levels of target genes MCOLN1 and RAB3A in HGC‐27 and AGS cells stably transfected with mock, *HOXA1*, scramble shRNA (sh‐Scb), or sh‐HOXA1 #1, and those co‐transfected with sh‐PITX2 #1 or *PITX2*. Data are shown as representative of three independent experiments in B‐F.

For investigating the binding partners critical for PITX2 function in gastric cancer, we conducted a comprehensive analysis of publicly available databases, including HuRI (http://www.interactome‐atlas.org/), InBioMap (https://inbio‐discover.com/), BioGRID (https:// thebiogrid.org/), and IID (http://iid.ophid.utoronto.ca/). This analysis revealed three consistent interactors of PITX2, two of which were linked to poor survival outcomes in gastric cancer patients, as evidenced by dataset from the Kaplan‐Meier Plotter database (**Figure** **4**A; Table S4, Supporting Information). Validation through co‐immunoprecipitation (co‐IP) followed by protein immunoblotting confirmed that PITX2 endogenously interacted with HOXA1, but not with beta‐catenin (CTNNB1), in HGC‐27 cells (Figure 4B). Notably, SD treatment enhanced the interaction between PITX2 and HOXA1 in HGC‐27 and SNU‐1 cells (Figure S7A, Supporting Information). Immunofluorescence analysis revealed that PITX2 and HOXA1 exhibited nuclear co‐localization in HGC‐27 cells, whereas forced expression of either protein promoted this interaction (Figure 4C). Using purified glutathione S‐transferase (GST)‐tagged HOXA1 and maltose binding protein (MBP)‐tagged PITX2 proteins, co‐IP followed by western blot analysis revealed that the first 83 amino acids of HOXA1 were essential for its interaction with PITX2 (Figure 4D). Additionally, the N‐terminal transactivation domain (N‐ter, 1‐38 amino acids) of PITX2, rather than its homeodomain (HD) or otp/aristaless/rax (OAR) domain, was crucial for binding to HOXA1 (Figure 4D). This finding was further corroborated in AGS cells expressing HA‐tagged *HOXA1* and FLAG‐tagged *PITX2* truncation constructs (Figure S7B, Supporting Information). A bimolecular fluorescence complementation (BiFC) assay confirmed the physical interaction of HOXA1 with PITX2 in AGS cells expressing their respective constructs (Figure 4E). Importantly, modulation of *HOXA1* levels via forced over‐expression or shRNA‐mediated knockdown significantly influenced the enrichment and activity of PITX2, expression of *MCOLN1* and *RAB3A*, and secretion of LGALS1 and IGFBP7 in HGC‐27, SNU‐1, AGS, or MKN‐45 cells with steady transfection of sh‐PITX2 #1 or *PITX2* (Figures S7C,D and S8A,B, Supporting Information and Figure 4F). Furthermore, forced expression or silencing of *HOXA1* reversed the reduction or accumulation of LAMP1 within PM in HGC‐27 or AGS cells with stable *PITX2* silencing or over‐expression (Figures S7E and S8C,D, Supporting Information). These findings indicated that HOXA1 interacted with PITX2 to promote lysosomal exocytosis‐related gene expression in gastric cancer cells.

### 2.5 PITX2 and HOXA1 Form Liquid Condensates in Gastric Cancer Cells

**Figure 5:**
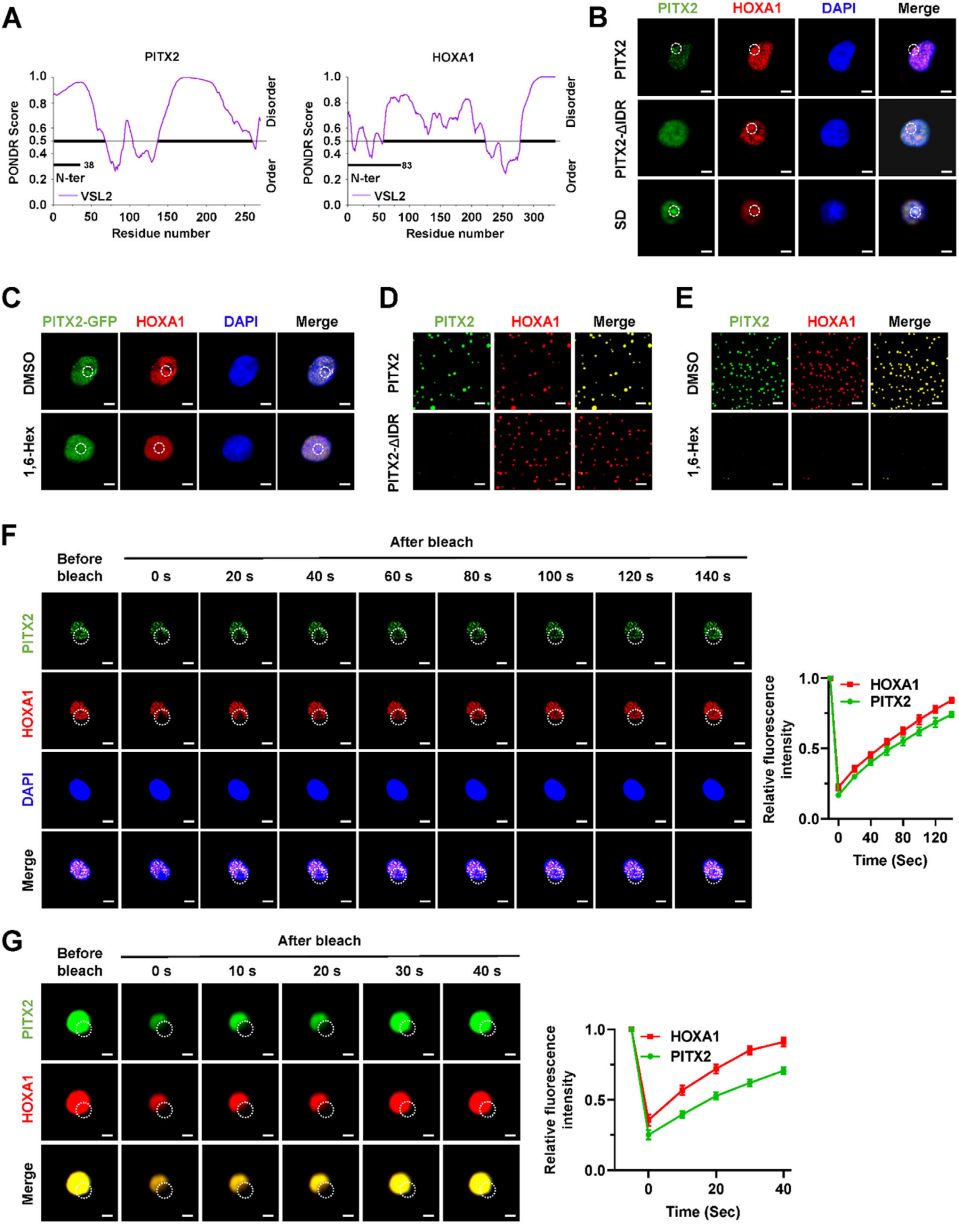
PITX2 and HOXA1 form liquid condensates in gastric cancer cells. A) IDRs within PITX2 and HOXA1 proteins analyzed by PONDR (http://www.pondr.com/) program. B) Fluorescence imaging assay indicating the condensate formation (circles) of EGFP‐PITX2 and HOXA1 in AGS cells stably transfected with wild‐type or IDR deficient (∆IDR) *PITX2* construct, and those treated with SD. Scale bars: 10 µm. C) Fluorescence imaging assay indicating the condensate formation (circles) of EGFP‐PITX2 and HOXA1 in AGS cells treated with DMSO or 1.5% 1,6‐Hex. D) Representative images of droplet formation of wild‐type or IDR deficient (∆IDR) EGFP‐PITX2 and HOXA1‐mCherry in droplet formation buffer. Scale bars: 10 µm. E) Representative images of droplet formation of EGFP‐PITX2 and HOXA1‐mCherry in the presence or absence of 1.5% 1,6‐Hex. Scale bars: 10 µm. F) Representative images (left panel) and quantification (right panel) of FRAP assay showing the exchange kinetics (circles) of EGFP‐PITX2 and HOXA1 in AGS cells stably transfected with *PITX2* construct. Scale bars: 10 µm. G) Representative images (left panel) and quantification (right panel) of FRAP assay showing the exchange kinetics (circles) of EGFP‐PITX2 and HOXA1‐mCherry proteins within condensates. Scale bars: 10 µm. Data are shown as representative of three independent experiments in B‐G.

The intrinsic disordered regions (IDRs) within HOXA1 and PITX2 proteins were identified via Predictor of Natural Disordered Regions (PONDR) program^[^ ^23^ ^]^ (**Figure** **5**A). To explore their capacity for liquid–liquid phase separation (LLPS), we observed the formation of nuclear puncta in AGS gastric cancer cells expressing EGFP‐tagged PITX2 or endogenous HOXA1, which were augmented under SD condition (Figure 5B). Deletion of the PITX2 IDR disrupted its ability to form liquid condensates (Figure 5B). Furthermore, exposure to 1,6‐hexanediol (1,6‐Hex), a recognized LLPS inhibitor, reduced the liquid condensates of EGFP‐tagged PITX2 and HOXA1 in AGS cells (Figure 5C). In vitro, recombinant EGFP‐tagged PITX2 and mCherry‐tagged HOXA1 proteins (purity >90%) were analyzed for their phase separation properties. They formed droplets in vitro, a process that was abolished by the removal of PITX2 IDR or presence of 1,6‐Hex (Figure 5D,E). To examine their liquid‐like characteristics, fluorescence recovery after photobleaching (FRAP) assay was conducted, revealing rapid recovery of intracellular EGFP‐tagged PITX2 and HOXA1 (Figure 5F) and their recombinant proteins in vitro (Figure 5G). Importantly, stable over‐expression of *HOXA1* or *PITX2* enhanced the PITX2 enrichment on *MCOLN1* or *RAB3A* promoter region, its transcriptional activity, and expression levels of *MCOLN1* or *RAB3A*, leading to increased lysosomal exocytosis of LGALS1 and IGFBP7, which was mitigated by 1,6‐Hex treatment (Figure S9A–C, Supporting Information). These findings demonstrated that HOXA1 and PITX2 formed liquid condensates in gastric cancer cells.

### 2.6 HOAX1/PITX2 Liquid Condensates Promote Gastric Cancer Progression Via Lysosomal Exocytosis

**Figure 6:**
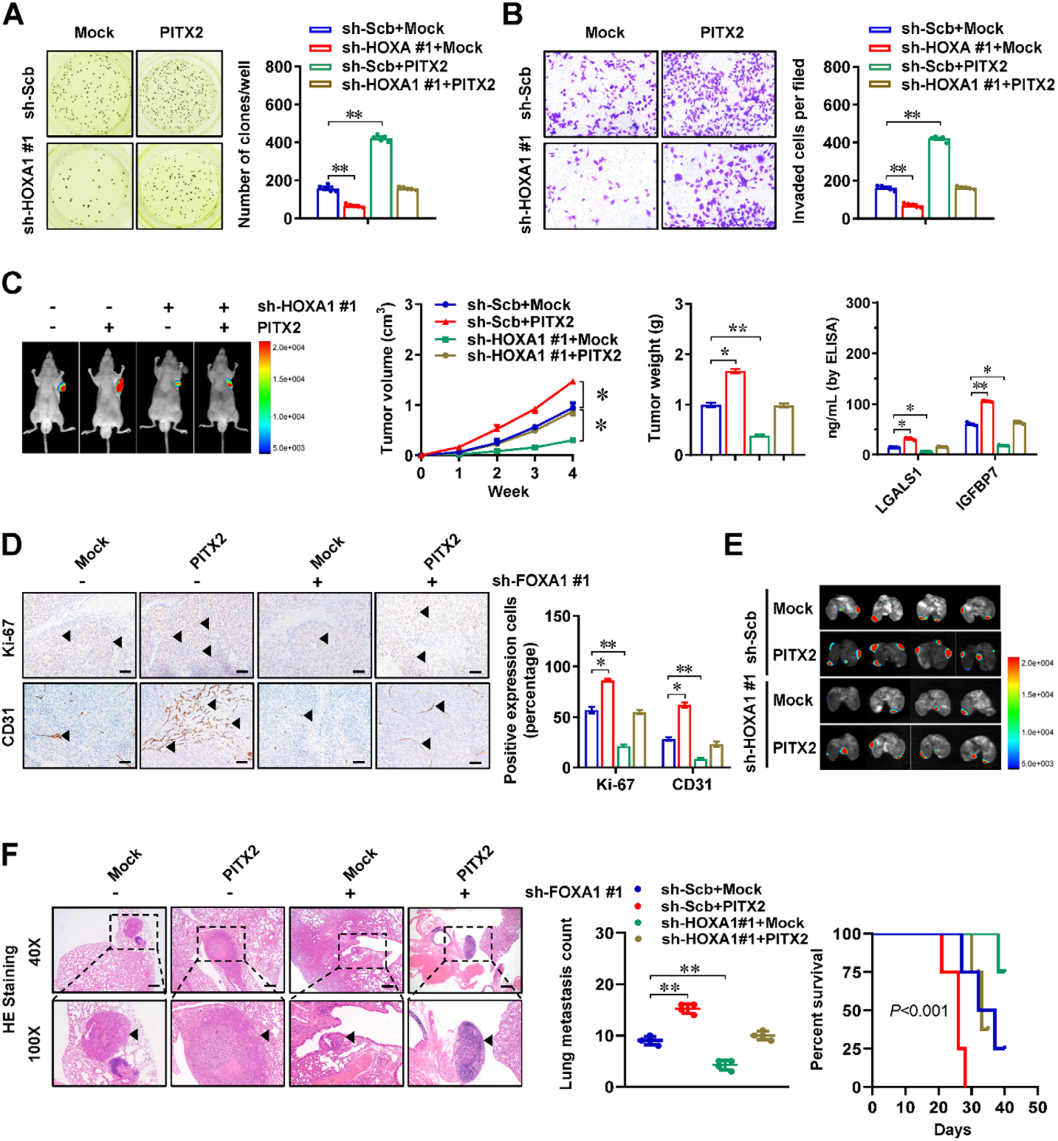
HOAX1/PITX2 liquid condensates promote gastric cancer progression via lysosomal exocytosiss. A and B) Representative images (left panel) and quantification (right panel) of soft agar (A) and matrigel invasion (B) assays indicating anchorage‐independent growth and invasion capability of MKN‐45 cells treated by culture medium form senescent cells stably transfected with scramble shRNA (sh‐Scb) or sh‐HOXA1 #1, and those co‐transfected with empty vector (mock) or *PITX2* (*n *= 5). C and D) Representative images (C, left panels), growth curve (C, middle panel), weight at the end points (C, middle panel), LGALS1 or IGFBP7 secretion (C, right panel), and immunohistochemical staining of Ki‐67 and CD31 (D, arrowheads) within tumor xenografts formed by subcutaneous injection of MKN‐45 cells in nude mice that subsequently treated with intravenous injection of culture medium collected from senescent cells stably transfected with sh‐Scb or sh‐HOXA1 #1, and those co‐transfected with mock or *PITX2* (*n *= 5 for each group). Scale bars: 100 µm. E and F) Representative images (E), hematoxylin‐eosin (HE) staining (F, arrowheads), quantification of lung metastatic colonization (F), and Kaplan‐Meier curves (F) of nude mice (*n *= 4 for each group) receiving vein tail injection of MKN‐45 cells that subsequently treated with intravenous injection of culture medium collected from senescent cells stably transfected with sh‐Scb or sh‐HOXA1 #1, and those co‐transfected with mock or *PITX2*. One‐way ANOVA compared the difference in A‐D and F. Log‐rank test for survival comparison in F. Data are shown as mean ± s.e.m. (error bars); ^*^, *p *<0.05; ^**^, *p *<0.01.

We further conducted rescue experiments to explore the functional interplay between *HOXA1* and *PITX2* in lysosomal exocytosis and aggressiveness of gastric cancer. The medium supernatant from senescent gastric cancer cells (MKN‐45 and HGC‐27) stably over‐expressing *PITX2* or sh‐PITX2 #1 was collected to induce the increase or decrease in anchorage‐independent growth and invasive properties of cancer cells, which were rescued by stable knockdown or over‐expression of *HOXA1* (**Figure** **6**A,B; Figure S9D,E, Supporting Information). The medium supernatant from MKN‐45 cells stably transfected with *PITX2* under SD condition was administered via tail vein to nude mice that had undergone subcutaneous injection of cancer cells. This led to increased growth, weight, LGALS1 and IGFBP7 secretion, Ki‐67 proliferation index, as well as CD31‐positive microvessels in xenograft tumors, accompanied by alterations in AKT phosphorylation and EMT markers, which were inhibited by *HOXA1* silencing (Figure 6C,D; Figure S9F, Supporting Information). Administering medium supernatant derived from senescent cancer cells stably over‐expressing *PITX2* via tail vein injection resulted in increase of lung metastasis and decrease of survival rates in nude mice injected with MKN‐45 cells (Figure 6E,F). Notably, this effect was mitigated by *HOXA1* knockdown (Figure 6E,F). Compared to their para‐tumoral tissues, higher levels of *HOXA1*, *PITX2*, *MCOLN1*, and *RAB3A* were observed in gastric cancer specimens (Figure S10A, Supporting Information). Kaplan‐Meier survival analysis revealed that elevated *HOXA1* levels were associated with worse overall (*P *= 5.3 × 10^−3^) and event‐free (*P *= 8.0 × 10^−3^) survival of gastric cancer patients (Figure S10B, Supporting Information). These findings suggested that HOXA1/PITX2 liquid condensates promoted gastric cancer progression via lysosomal exocytosis.

### 2.7 Nortriptyline Inhibits the Interaction and Phase Separation of HOXA1 and PITX2 in Gastric Cancer

**Figure 7:**
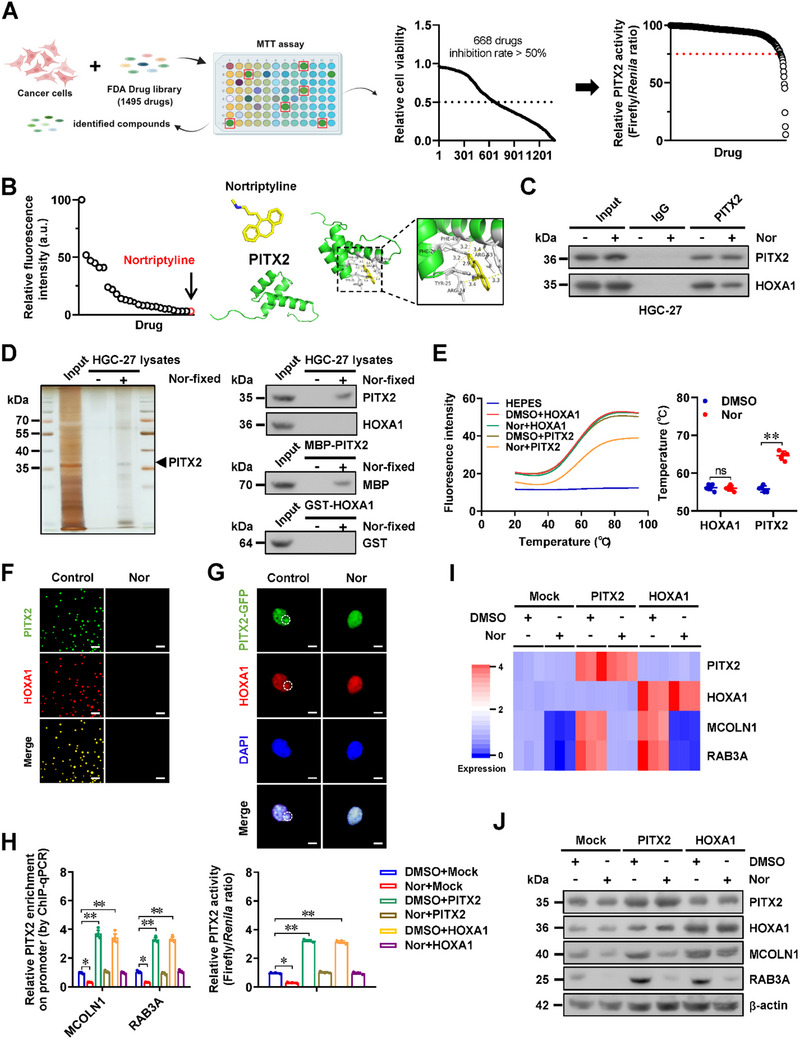
Nortriptyline inhibits the interaction and phase separation of HOXA1 and PITX2 in gastric cancer cells. A) Schematic illustration (left panel), MTT colorimetric assay (middle panel), and dual‐luciferase assay (right panel) indicating the screening of PITX2 activity inhibitor from 1495 FDA‐approved drugs. B) BiFC assay using VN173‐HOXA1 and VC155‐PITX2 constructs (left panel) and molecular docking (right panel) via CB‐Dock2 program (http://183.56.231.194:8001/cb‐dock2) showing the screening of compounds repressing the HOXA1‐PITX2 interaction, as well as potential interaction of nortriptyline with PITX2 protein. C) Validating co‐IP and western blot assays indicating the binding of PITX2 to HOAX1 in HGC‐27 cells treated with DMSO or nortriptyline (Nor, 40 µmol·L^−1^). D) Silver staining (left panel) and western blot (right panel) assays indicating affinity of proteins within HGC‐27 cell lysates or recombinant proteins with FG beads covalently conjugated with Nor (40 µmol·L^−1^). E) DSF assay and temperature showing the fluroresence intensity of HEPES or recombinant HOXA1 or PITX2 protein treated with solvent (DMSO) or Nor (40 µmol·L^−1^, *n *= 5). F) Representative images of droplet formation of EGFP‐PITX2 and HOXA1‐mCherry proteins incubated with vehicle or Nor (40 µmol·L^−1^). Scale bar: 5 µm. G) Fluorescence imaging assay indicating the condensate formation (circles) of EGFP‐PITX2 and HOXA1 in AGS cells stably transfected with *PITX2* construct, and those treated with vehicle or Nor (40 µmol·L^−1^). Scale bars: 10 µm. H) ChIP‐qPCR (normalized to input, *n *= 3) and dual‐luciferase reporter (*n *= 3) assays showing the PITX2 enrichment on *MCOLLN1* or *RAB3A* promoter regions as well as PITX2 activity in MKN‐45 cells stably transfected with empty vector (mock), *PITX2*, or *HOXA1*, and those treated with vehicle or Nor (40 µmol·L^−1^). I and J) Heatmap of real‐time qRT‐PCR (I, normalized to *β‐actin*, *n *= 5) and western blot (J) assays indicating the transcript and protein levels of *MCOLLN1* and *RAB3A* in MKN‐45 cells stably transfected with mock, *PITX2*, or *HOXA1*, and those treated with vehicle or Nor (40 µmol·L^−1^). Student's t‐test or one‐way ANOVA compared the difference in E and H. Data are shown as mean ± s.e.m. (error bars); ^*^, *p *<0.05; ^**^, *p *<0.01; ns, non‐significant.

For discovering inhibitors of HOXA1‐PITX2 interaction, we employed a high‐throughput screening approach using 1495 Food and Drug Administration (FDA)‐approved drugs (**Figure** **7**A). Through a tetrazolium bromide (MTT) colorimetric assay, 668 compounds with cellular viability inhibition rates exceeding 50% were selected (Figure 7A). These compounds were further evaluated in a dual‐luciferase reporter study to reveal that 25 compounds were capable of suppressing PITX2 activity by more than 25% (Figure 7A). Among them, promethazine HCl, clozapine, lubiprostone, dihydroergotamine mesylate, fluphenazine dihydrochloride, perphenazine, nortriptyline (Nor), and donepezil HCl were identified as inhibitors of HOXA1‐PITX2 interaction in a BiFC assay, with Nor exhibiting the strongest inhibitory effect (Figure 7B; Figure S11A, Supporting Information). Molecular docking analysis was performed using PDB files from RCSB PDB (https://www.rcsb.org) and Drug Bank (https://go.drugbank.com), alongside the CB‐Dock2 program (http://183.56.231.194:8001/cb‐dock2).^[^ ^24^ ^]^ To explore common error margin of approximately 2 kcal mol^−1^ in binding energy due to inherent stochasticity of algorithms,^[^ ^25^ ^]^ five replicates of molecular docking were carried out to indicate the direct interaction between PITX2 and Nor (AutoDock Vina score = − 81.3 ± −1.33 kcal mol^−1^), with a minimum distance of 2.9 Å (Figure 7B). Co‐IP and western blot analyses revealed that Nor effectively inhibited the interaction of PITX2 with HOXA1 (Figure 7C). Affinity purification using ferrite glycidyl methacrylate (FG) beads, followed by silver staining and western blot assays, confirmed that Nor directly bound to PITX2 protein within HGC‐27 cell lysates or recombinant PITX2 protein, but not to HOXA1 protein (Figure 7D). Furthermore, differential scanning fluorimetry (DSF) was used to assess the impact of Nor on the thermal stability of recombinant PITX2 and HOXA1 proteins. The results indicated that Nor treatment reduced the thermal stability of PITX2 rather than HOXA1 (Figure 7E). Additionally, Nor treatment significantly disrupted the liquid condensates of HOXA1 and PITX2 both in vitro and in vivo (Figure 7F,G) and abolished the increase in enrichment and activity of PITX2, as well as expression of *MCOLN1* or *RAB3A*, in MKN‐45 cells with stable *PITX2* or *HOXA1* over‐expression (Figure 7H–J). These findings indicated that nortriptyline inhibited the interaction and phase separation of HOXA1 and PITX2 in gastric cancer.

### 2.8 Nortriptyline Suppresses Lysosomal Exocytosis and Progression of Gastric Cancer

**Figure 8:**
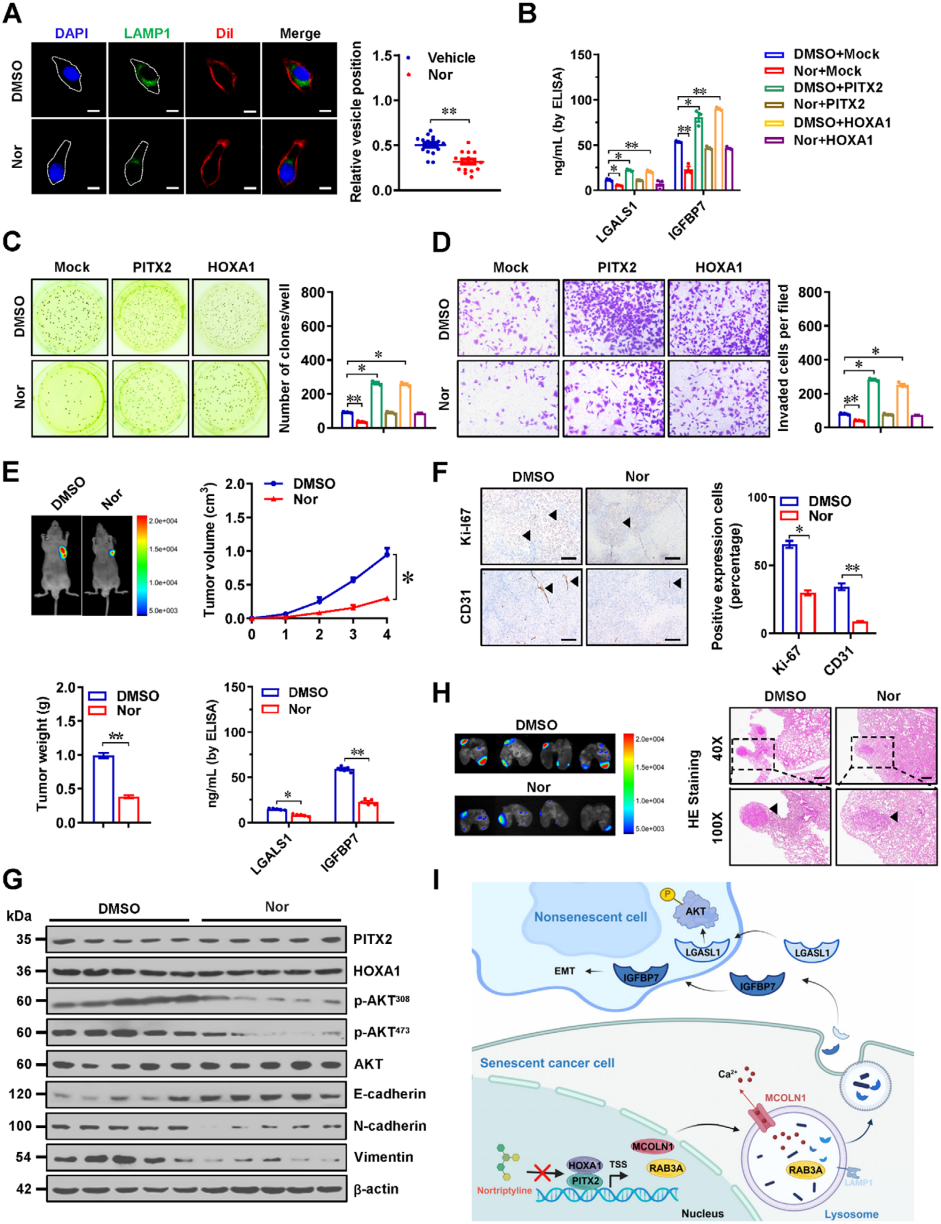
Nortriptyline suppresses lysosomal exocytosis and progression of gastric cancer. A) Representative images (left panel) and quantification (right panel) of LAMP1 and Dil staining in HGC‐27 cells treated with vehicle or Nor (40 µmol·L^−1^). Scale bars: 10 µm. B) ELISA showing the levels of LGALS1 and IGFBP7 within culture medium of MKN‐45 cells stably transfected with empty vector (mock), *PITX2*, or *HOXA1*, and those treated with vehicle or Nor (40 µmol·L^−1^, *n *= 3). C and D) Representative images (left panel) and quantitative (right panel) of soft agar (C) and matrigel invasion (D) assays showing the growth and invasion of MKN‐45 cells treated with culture medium from those stably transfected with mock, *PITX2*, or *HOXA1*, and those treated with vehicle or Nor (40 µmol·L^−1^, *n *= 3). E and F) Representative images (E), growth curve (E), weight at the end points (E), LGALS1 or IGFBP7 secretion (E, right panel), and immunohistochemical staining of Ki‐67 and CD31 (F, arrowheads) within tumor xenografts formed by subcutaneous injection of HGC‐27 cells in nude mice that subsequently treated with Nor (50 mg·kg^−1^, *n *= 5 for each group). Scale bars: 100 µm. G) Western blot assay showing the expression levels of PITX2, HOAX1, p‐AKT^308^, p‐AKT^473^, E‐cadherin, N‐cadherin, or vimention in xenograft tumors formed by subcutaneous injection of HGC‐27 cells in nude mice that subsequently treated with Nor (50 mg·kg^−1^, *n *= 5 for each group). H) Representative images and hematoxylin‐eosin (HE) staining of lung metastatic colonization (arrowheads) of nude mice (*n *= 4 for each group) receiving vein tail injection of HGC‐27 cells and subsequent intravenous administration of Nor (50 mg·kg^−1^). I) Schematic depicting the mechanisms underlying *HOAX1*/*PITX2*‐regulated cancer progression: as a transcription factor, PITX2 facilitates the expression of lysosomal exocytosis genes (*MCOLN1* and *RAB3A*), while HOXA1 interacts with PITX2 to facilitate its activity, resulting in increase of lysosomal exocytosis‐mediated SASP (LGASL1 or IGFBP7) and subsequent activation of AKT signaling and EMT. Meanwhile, nortriptyline (Nor) is able to block HOXA1‐PITX2 interaction, resulting in inhibition of lysosomal exocytosis, tumorigenesis, and aggressiveness. Student's *t* test or one‐way ANOVA compared the difference in A‐F. Data are shown as mean ± s.e.m. (error bars); ^*^, *p *<0.05; ^**^, *p *<0.01.

To investigate the therapeutic potential of Nor in targeting lysosomal exocytosis and progression of gastric cancer, we conducted a series of experiments. Confocal imaging and ELISA assays demonstrated that Nor treatment effectively inhibited lysosomal exocytosis in HGC‐27 cells (**Figure** **8**A), and reduced the secretion of LGALS1 and IGFBP7 in MKN‐45 cells stably over‐expressing *PITX2* or *HOXA1* (Figure 8B). Functional assays, including soft agar and matrigel invasion experiments, indicated that Nor treatment significantly attenuated the increase in proliferative and invasive capabilities of MKN‐45 cells over‐expressing *PITX2* or *HOXA1* (Figure 8C,D). Furthermore, in vivo administration of Nor led to a reduction in tumor growth, weight, and secretion of LGALS1 and IGFBP7 in HGC‐27‐formed xenograft models, alongside decreased proliferation index (Ki‐67), reduced CD31‐positive microvessels, and altered levels of AKT phosphorylation and EMT markers (Figure 8E–G). Intravenous administration of Nor also led to fewer lung metastasis and improved survival of nude mice that intravenously injected with HGC‐27 cells (Figure 8H; Figure S11B, Supporting Information). These findings collectively indicated that Nor suppressed lysosomal exocytosis and progression of gastric cancer.

## 3 Discussion

Senescent cells have a range of core characteristics, including persistent growth arrest, senescence‐related gene expression,^[^ ^26^ ^]^ and secretion of a complex mixture termed as SASP.^[^ ^27^ ^]^ However, the contribution of lysosomal exocytosis to SASP production in gastric cancer remains poorly understood. Herein, our findings revealed the critical roles of HOXA1 and its interacting PITX2 in lysosomal exocytosis‐mediated SASP via phase‐separated biomolecular condensates (Figure 8I). Bio‐molecular condensates are dynamic and membraneless assemblies arising through LLPS, and control cellular processes by concentrating specific biomolecules (such as transcriptional regulators) into distinct compartments.^[^ ^28^ ^]^ For instance, far upstream element binding protein 3 undergoes phase separation to form liquid‐like condensates, which facilitate the activity of suppressor of zest 12 during tumor progression.^[^ ^29^ ^]^ Our data indicated that HOXA1 cooperated with PITX2 to possess oncogenic properties in lysosomal exocytosis of contents (LGALS1 and IGFBP7), resulting in activation of AKT signaling and EMT, tumorigenesis, and aggressiveness. We also found that *PITX2* facilitated or repressed the lysosomal exocytosis of many SASP proteins, including MMP2, IGFBP2, and TIMP2, in senescent gastric cancer cells. Although IL‐6 and interleukin 18 were detected as SASP proteins, their levels were not affected by *PITX2*‐mediated lysosomal exocytosis, suggesting the involvement of alternative mechanisms in regulating SASP in gastric cancer. Of notes, *LGALS1* drives αvβ3‐integrin expression to activate AKT signaling pathway essential for EMT in hepatocellular carcinoma.^[^ ^21^ ^]^ Meanwhile, *IGFBP7* is up‐regulated in gastric cancer to promote EMT by increasing the levels of N‐cadherin, Snail, and Vimentin while decreasing E‐cadherin expression.^[^ ^22^ ^]^ *IGFBP7* is also highly expressed in cancer‐associated fibroblasts and mesenchymal cells, which promotes M2 macrophage activation and gastric cancer progression via fibroblast growth factor 2/fibroblast growth factor receptor 1/PI3K/AKT axis.^[^ ^30^ ^]^ Considering the intersection of AKT signaling and EMT process in multiply biological processes, further investigation is warranted to reveal the functions and therapeutic targeting of *PITX2*‐mediated lysosomal exocytosis of LGALS1 and IGFBP7 in senescence surveillance, immune evasion, or senescence surveillance.

PITX2, a highly conserved transcription factor of bicoid homeodomain family, plays diverse roles in embryonic development of eyes, teeth, and abdominal viscera.^[^ ^31^ ^]^ Recent studies show the elevation of *PITX2* expression in multiple cancer types, including lung adenocarcinoma (LUAD),^[^ ^32^ ^]^ colorectal cancer,^[^ ^33^ ^]^ ovarian malignancies,^[^ ^34^ ^]^ and esophageal squamous cell carcinoma,^[^ ^35^ ^]^ which is linked to advanced stages and poor prognosis.^[^ ^32^ ^]^ Conversely, in prostate cancer, reduced *PITX2* expression is associated with poor clinical outcomes,^[^ ^36^ ^]^ suggesting its context‐dependent roles in tumorigenesis. In LUAD, *PITX2* promotes transcription of *WNT3A* to activate Wnt/β‐catenin signaling pathway, while silencing *PITX2* significantly inhibits the proliferative and metastatic capacities of lung cancer cells.^[^ ^32^ ^]^ *PITX2* over‐expression enhances the invasiveness of ovarian cancer via activation of transforming growth factor beta signaling pathway and activin A.^[^ ^34^ ^]^ *PITX2* also contributes to doxorubicin resistance in renal cancer cells through modulation of ATP‐binding cassette subfamily B member 1 expression.^[^ ^33^ ^]^ In our study, we observed that PITX2 significantly up‐regulated the transcription of *MCOLN1* and *RAB3A*. MCOLN1, one member of the transient receptor potential superfamily, mediates the release of divalent cations (such as Ca^2^⁺, Zn^2^⁺, and Fe^2^⁺), and plays an integral role in lysosomal exocytosis, fusion, trafficking, or autophagy.^[^ ^10^ ^]^ Although initial studies propose that *MCOLN1*‐mediated Ca^2^⁺ release promotes autophagy via transcription factor EB activation,^[^ ^17a^ ^]^ accumulating evidences indicate that it disrupts autophagosome‐lysosome fusion via inducing lysosomal Zn^2^⁺ efflux and interfering with the complex of syntaxin 17 (STX17) and vesicle associated membrane protein 8 (VAMP8) in pancreatic cancer, breast cancer, gastric cancer, malignant melanoma, and glioblastoma.^[^ ^17^, ^38^ ^]^ In addition, *MCOLN1* promotes AKT‐driven tumorigenesis and therapeutic resistance by inhibiting ferroptosis.^[^ ^39^ ^]^ As one member of trafficking molecular machinery partially localizing at peripheral lysosomes, *RAB3A* is essential for lysosome exocytosis, while its knockdown results in positioning of lysosomes to perinuclear region.^[^ ^16b^ ^]^ Our studies showed that *PITX2* promoted lysosomal exocytosis of senescent gastric cancer cells, along with arrest of autophagosome‐lysosome fusion, via regulating *MCOLN1* expression. Consistent with the functions of *ATG5* in lysosomal exocytosis,^[^ ^18^ ^]^ we found that its knockdown led to enhancement of lysosomal exocytosis in gastric cancer cells, implicating the regulatory interplay between autophagy and lysosomal exocytosis, while the underlying mechanisms deserve further studies.

HOXA1, a member of the HOX gene family originally identified in *Drosophila*, encodes a DNA‐binding transcription factor that regulates cellular differentiation, morphogenesis, and proliferation.^[^ ^40^ ^]^ *HOXA1* is over‐expressed in multiple cancers, including breast, lung, liver, prostate or gastric cancer, and its elevated expression is associated with advanced cancer progression and unfavorable clinical outcomes.^[^ ^41^ ^]^ In breast cancer, *HOXA1* promotes oncogenic transformation by activating p44/42 MAP kinase pathway, and is associated with endocrine therapy resistance.^[^ ^42^ ^]^ In gastric cancer, *HOXA1* significantly contributes to EMT via the PI3K/AKT signaling pathway, while it drives aerobic glycolysis to facilitate tumor progression in cervical cancer.^[^ ^43^ ^]^ In contrast, *HOXA1* down‐regulation is correlated with negative clinical outcomes in small cell lung cancer,^[^ ^44^ ^]^ highlighting its context‐dependent function. In this study, we found that 1‐83 amino acids of HOXA1 was able to interact with N‐terminal transactivation domain of PITX2, resulting in enhanced PITX2 activity to drive transcription of *MCOLN1* and *RAB3A* in gastric cancer cells. Our data demonstrated that HOXA1 facilitated the progression of gastric cancer, at least in part, via its interaction with PITX2, indicating the pivotal functions of *HOXA1*/*PITX2* axis in the progression of gastric cancer.

In recent years, targeting SASP has been recognized as a promising cancer treatment strategy. Depleting senescent hepatic stellate cells by dasatinib/quercetin or ABT‐263 is able to inhibit tumor progression.^[^ ^45^ ^]^ SASP can be also regulated by glucocorticoids, metformin, or Janus kinase inhibitors.^[^ ^46^ ^]^ In this study, we found that nortriptyline blocked the interaction between HOXA1 and PITX2. Nortriptyline, a classic tricyclic antidepressant, is commonly prescribed for depression through its inhibition of serotonin and norepinephrine reuptake in neuronal membranes.^[^ ^47^ ^]^ Interestingly, nortriptyline exerts potent antineoplastic activity across multiple cancer types, including bladder cancer,^[^ ^48^ ^]^ prostate cancer,^[^ ^49^ ^]^ and lung cancer.^[^ ^50^ ^]^ Nortriptyline decreases the expression of RNA binding motif single stranded interacting protein 1 (*RBMS1*) to sensitize lung cancer cells for radiotherapy.^[^ ^50^ ^]^ In bladder cancer, nortriptyline increases reactive oxygen species production and induces intrinsic or extrinsic apoptosis.^[^ ^48^ ^]^ In pineoblastoma, nortriptyline destroys lysosomes and inhibits tumor proliferation.^[^ ^51^ ^]^ In this investigation, we demonstrated that nortriptyline suppressed the activity of PITX2 and its downstream gene expression, resulting in reduction in lysosomal exocytosis‐mediated SASP, tumorigenic potential, and invasive behavior of gastric cancer cells, highlighting its potential values in senomorphic intervention. Of note, mining of DINIES (https://www.genome.jp/tools/ dinies/),^[^ ^52^ ^]^ a drug‐target interaction database, reveals additional protein partners of nortriptyline, such as its metabolic enzyme cytochrome P450 family 3 subfamily A member 4 (CYP3A4),^[^ ^53^ ^]^ lamin A/C, and monoamine transporters.^[^ ^54^ ^]^ Thus, future research is needed to explore the impact of nortriptyline on expression profiles (eg. LLPS) or functions of other protein partners, and elucidate its potential off‐target effects in cancer therapy.

In summary, our findings reveal that *PITX2* is significantly linked to unfavorable outcomes in gastric cancer and modulates the expression of lysosomal exocytic genes, including *MCOLN1* and *RAB3A*, which enhance the secretion of LGALS1 and IGFBP7. These factors are essential for the SASP production, tumorigenesis, and metastasis. Furthermore, HOXA1 interacts with PITX2, increasing its activity levels and thereby promoting lysosomal exocytosis‐mediated SASP associated with cancer aggressiveness. Interestingly, treatment with nortriptyline disrupts the HOXA1‐PITX2 interaction, effectively reducing tumor growth and invasiveness. This study highlights the essential role of HOXA1‐PITX2 axis in the regulation of lysosomal exocytosis and suggests innovative therapeutic approaches for cancer treatment.

## 4 Experimental Section

### Cell Lines

Gastric carcinoma cell lines NCI‐N87 (CRL‐5822), MKN‐28 (JCRB0253), MKN‐45 (JCRB0254), SNU‐1 (CRL‐5971), AGS (CRL‐1739), and HGC‐27 (CL‐0107), cervical carcinoma HeLa (CCL‐2), prostate cancer PC‐3 (CRL‐1435), and embryonic kidney HEK293T (CRL‐11268) cells, were acquired from the American Type Culture Collection (ATCC, Manassas, VA) and Japanese Collection of Research Bioresources (JCRB, Tokyo, Japan). Cellular identity was verified through short tandem repeat (STR) genotyping, with experiments conducted within six months of thawing frozen stocks. Routine mycoplasma screening was performed using MycoAlert™ Mycoplasma Detection Kit (4460623, Thermo Fisher Scientific, Waltham, MA). Cells were propagated in a humidified incubator at 37°C and 5% CO_2_, using RPMI 1640 medium prepared with 10% fetal bovine serum (Sigma, St. Louis, MO), and treated as specified with vacuolin‐1 (673000, Sigma), ionomycin (407951, Sigma), ML‐SI3 (MedChemExpress, Monmouth Junction, NJ), ML‐SA1 (MedChemExpress), 1,6‐hexanediol (88571, Sigma), or Nor (N‐907, Sigma).

### Western Blotting

Cellular subfractionation was conducted utilizing a commercial Fractionation Kit (9038, Cell Signaling Technology, Danvers, MA). Total protein isolation from tissues or cultured cells employed RIPA Buffer (89900, Thermo Fisher Scientific). Immunoblotting analysis was subsequently performed,^[^ ^2^, ^29^, ^55^ ^]^ using specific primary antibodies specific for PITX2 (ab98297, Abcam Inc., Cambridge, MA), MCOLN1 (92176S, Cell Signaling Technology), RAB3A (ab302518), P16 (ab51243), P21 (ab109520), LC3 (ab192890), SQSTM1 (ab109012), ATG5 (ab108327), LAMP1 (ab24170), Na^+^/K^+^‐ATPase (23565S), p‐Akt^308^ (13038), p‐Akt^473^(4060), AKT (ab8805), E‐cadherin (ab40772), N‐cadherin (ab76011), Vimentin (ab92547), HOXA1 (ab230513), CTNNB1 (9566, Cell Signaling Technology), GST‐tag (ab111947), MBP‐tag (2396, Cell Signaling Technology Inc.), HA‐tag (ab236632), FLAG‐tag (ab205606), GAPDH (ab8245), or β‐actin (ab6276). The original immunoblotting images were photographed directly by a ChemiDoc‐IT 610 Imaging System (UVP, Upland, CA) under consistent exposure settings, with identical total exposure time and parameters for all samples. For visual clarity and presentation consistency, the entire images were equally subjected to minor adjustments of brightness and contrast using Adobe Photoshop 18.0 software (Adobe Systems Inc., San Jose, CA), which was performed to faithfully represent the original data.

### ChIP‐qPCR

Cancer cells were seeded into 10‐cm culture dishes and subjected to ChIP assay using the EZ‐ChIP kit (MerkMillipore, Darmstadt, Germany).^[^ ^2^, ^29^, ^55^ ^]^ The assay was conducted with an antibody specific for PITX2 (ab221142). Real‐time quantitative PCR (qPCR) was undertaken with QuantiTect SYBR Green PCR Kit (204145, Qiagen, Hilden, Germany) and primers (Table S5, Supporting Information).

### RNA Sequencing (RNA‐Seq)

Cancer cells (1 × 10⁶) were lysed using RLT buffer (79216, Qiagen) combined with proteinase K digestion for RNA extraction with TRIzol^®^ (15596018, Thermo Fisher Scientific). Library preparation and transcriptome sequencing were performed using the BGISEQ‐500 platform (BGI Tech, Shenzhen, China). RNA‐seq reads of 100‐bp paired‐end were aligned to genes using Kallisto v0.44.0, with transcript abundance expressed as fragments per kilobase of transcript per million mapped fragments (FPKM). The raw sequencing data were deposited in the GEO database under accession number GSE305469.

### Co‐IP and Mass Spectrometry

Co‐IP was undertaken as documented previously,^[^ ^2^, ^29^, ^55^ ^]^using 10 µg of antibodies for PITX2 (ab221142), HOXA1 (ab230513), GST‐tag (ab111947), MBP‐tag (2396, Cell Signaling Technology Inc.), FLAG‐tag (ab205606), or HA‐tag (ab236632, Abcam Inc.). Following elution from the magnetic bead complexes, proteins were quantified using western blot analysis. For proteomic analysis, proteins were enzymatically digested with trypsin, and the resulting peptides were extracted. The LC‐MS/MS analysis was performed on a hybrid quadrupole‐TOF LC/MS/MS system (SCIEX, Redwood City, CA). Each acquisition cycle included a full‐scan mass spectrum (m/z range: 350–1500, charge states: 2–5) followed by 40 MS/MS fragmentation events. The results were processed via ProteinPilot Software v5.0 and searched against the UniProt human protein database for sequence identification.

### Cellular Viability, Growth, and Invasion Assays

Cellular viability, growth, and invasion in vitro was measured via the colorimetric assay with MTT (MerkMillipore),^[^ ^29^, ^55^ ^]^ soft agar assay,^[^ ^2^, ^29^, ^55^ ^]^ and matrigel invasion assay,^[^ ^29^, ^55^, ^56^ ^]^ respectively.

### Immunohistochemical Staining

Immunohistochemistry and quantitative analyses were carried out,^[^ ^2^, ^55^ ^]^ utilizing a monoclonal antibody targeting Ki‐67 (ab15580; diluted at 1:100) or another specific for CD31 (ab28364; diluted at 1:50).

### Statistical Analysis

The data were analyzed by GraphPad 8.0 software (GraphPad Software, Boston, MA). All data were expressed as mean ± standard deviation (SD). Cutoff thresholds were established based on the median or average gene expression levels. Statistical comparisons between groups were conducted using two‐tailed Student's t‐test (for normally distributed parametric data) or one‐way analysis of variance (ANOVA, for multiple distributed parametric data). The significance of overlaps was evaluated using Fisher's exact test. Kaplan‐Meier curves were applied for analyzing survival data, while group comparisons were performed using two‐sided log‐rank test. All statistical analyses were two‐tailed, with P‐values less than 0.05 considered statistically significant.

Detailed Experimental Section is described in the Supporting Information.

## Conflict of Interest

The authors declare no conflict of interest.

## Author Contributions

Y.Z., C.Y., X.L., and X.J.W. contributed equally to this work. Y.Z. and C.Y. conceived and designed the research. Y.Z., C.Y., X.L., X.J.W., W.J., X.L.W., J.Q., B.Z., S.Z., and performed the experiments. Y.Z. and C.Y. analyzed the data. Y.Z., Q.T., and L.Z. wrote the manuscript. All authors read and approved the final manuscript.

## Supporting information



Supporting Information

## Data Availability

The data that support the findings of this study are available in the supplementary material of this article.‐
