# Supplementary material for: Nortriptyline Inhibits Lysosomal Exocytosis‐Mediated SASP During Gastric Cancer Progression via Targeting HOXA1‐PITX2 Phase Separation
Source: Adv Sci (Weinh). 2025 Sep 25;12(46):e12407. doi: 10.1002/advs.202512407 (PMC12697896; doi:10.1002/advs.202512407)
Supplement: Supplementary file 1 — Supporting Information [file ADVS-12-e12407-s001.pdf]

Supporting Information

Nortriptyline Inhibits Lysosomal Exocytosis-Mediated SASP During Gastric Cancer Progression via Targeting HOXA1-PITX2 Phase Separation

Yi Zhou, *Chunhui Yang*, *Xinyue Li*, *Xiaojing Wang*, *Wanju Jiao*, *Xiaolin Wang*, *Jiaying Qu*, *Bosen Zhao*, *Shunchen Zhou*, *Qiangsong Tong* *, *Liduan Zheng* *

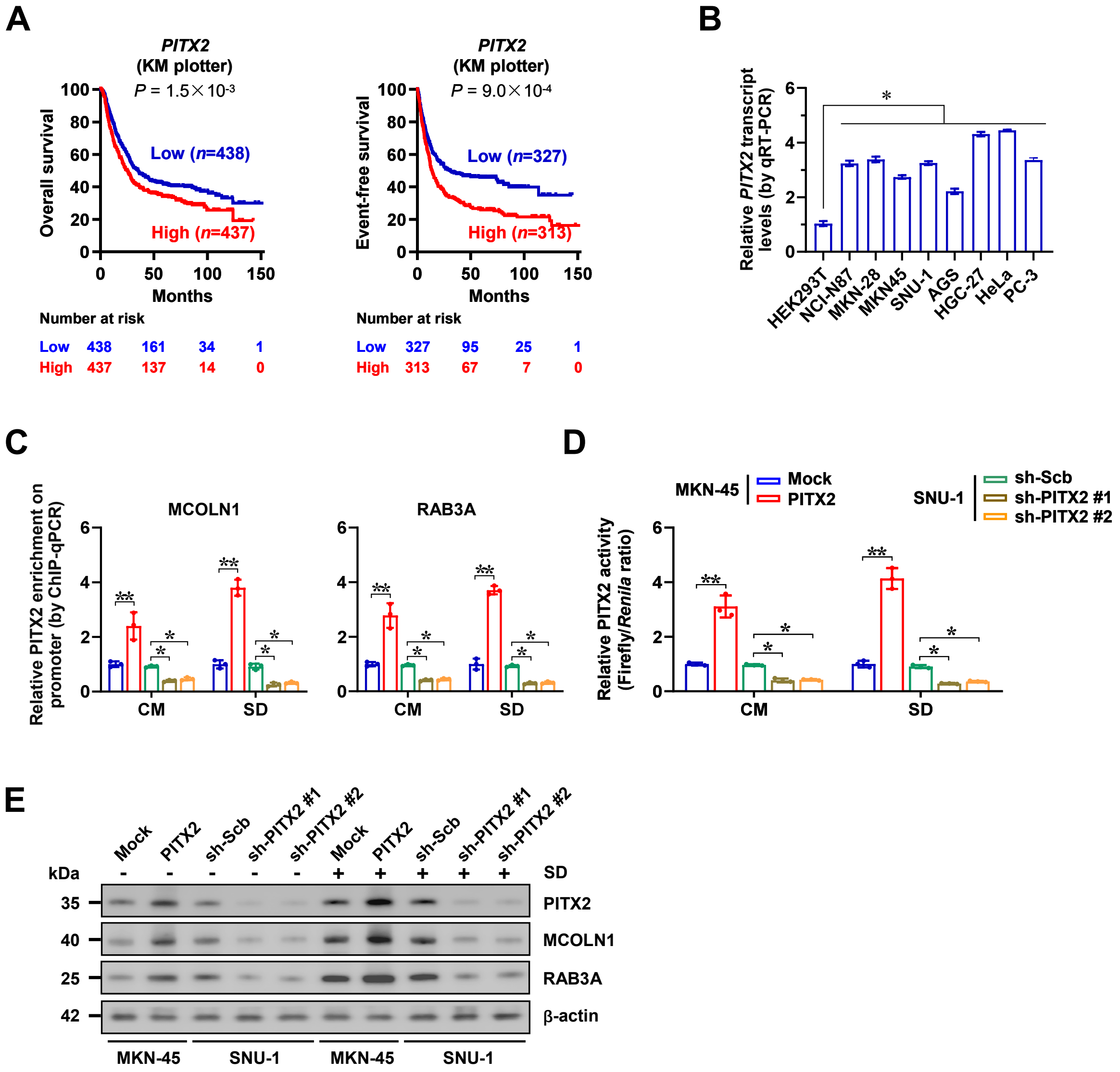

Figure S1. Expression profiles of *PITX2* and target genes in gastric cancer. A) Kaplan-Meier curves indicating overall and event-free survival of gastric cancer cases with low or high levels of *PITX2* (cutoff values=4.95 and 4.95). **B**) Real-time qRT-PCR assay indicating the levels (normalized to *β-actin*, *n*=5) of *PITX2* in HEK293T cells and cancer cell lines. **C**-**E**) ChIP-qPCR assay (C, normalized to input, *n*=3), dual-luciferase (D, *n*=3), and western blot (E) assays showing the PITX2 enrichment on target genes, PITX2 activity, and protein levels of MCOLLN1 and RAB3A in MKN-45 and SNU-1 cells stably transfected with empty vector (mock), *PITX2*, scramble shRNA (sh-Scb), sh-PITX2 #1, or sh-PITX2 #2 under CM or SD condition. Log-rank test for survival comparison in **A**. One-way ANOVA compared the difference in **B**-**D**. Data are shown as mean ± s.e.m. (error bars); *, P<0.05; **, P<0.01.

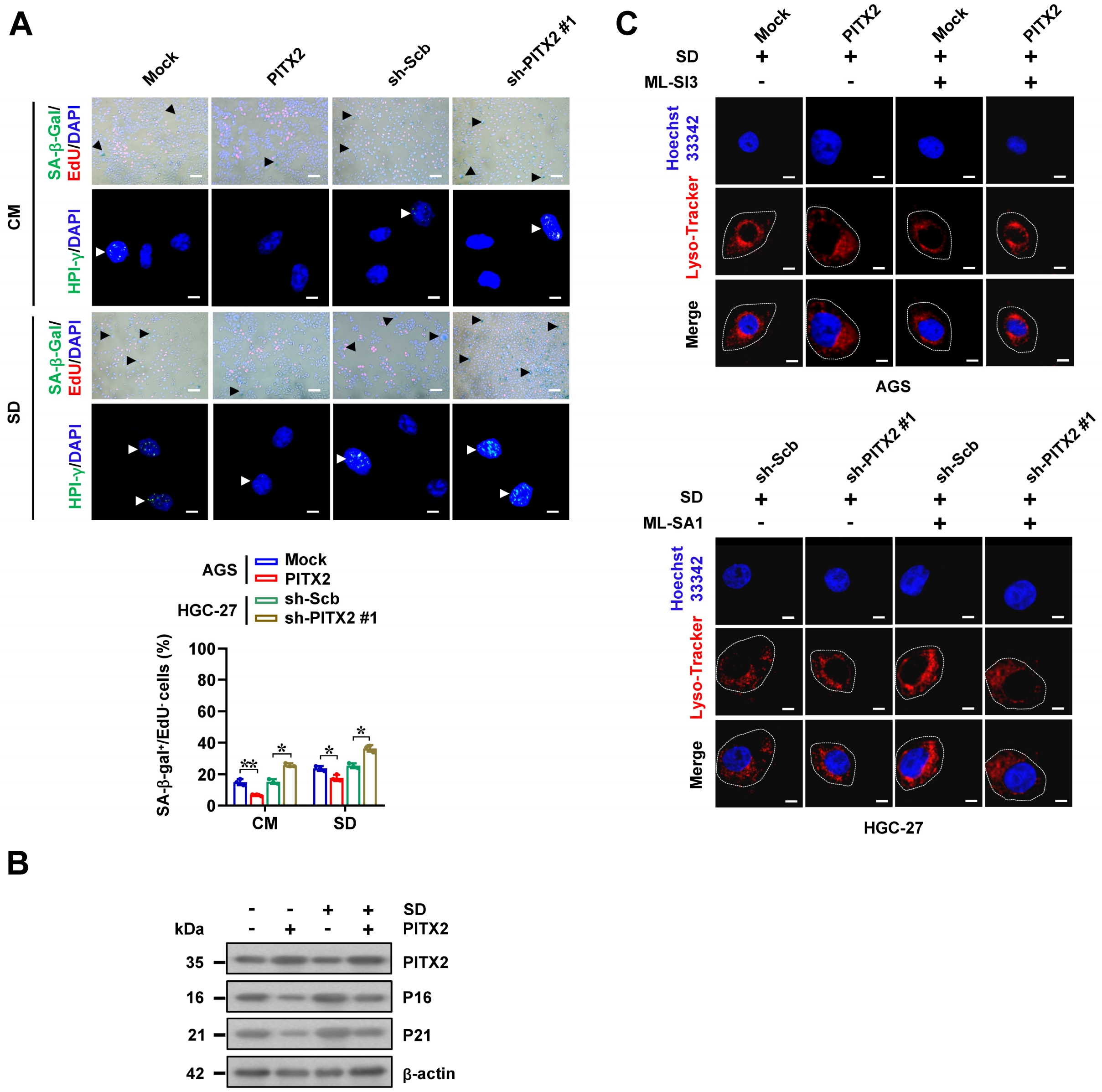

Figure S2. ***PITX2* promotes lysosomal exocytosis of gastric cancer cells. A**) Confocal images (upper panel) and quantification (lower panel) showing the SA-β-gal^{+}/EdU^{-} staining and HP-1γ-positive heterochromatic lesions (arrowheads) in AGS and HGC-27 cells stably transfected with empty vector (mock), *PITX2*, scramble shRNA (sh-Scb), or sh-PITX2 #1 under complete medium (CM) or serum deprivation (SD) conditions (n=3). Scale bars: 10 μm. **B**) Western blot assay indicating the levels of P16 and P21 in AGS cells stably transfected with mock or *PITX2* under prolonged SD condition for 10 days. **C**) Representative Lyso-Tracker Red staining images showing the number and anterograde transport distance of lysosomes in AGS and HGC-27 cells stably transfected with mock, *PITX2*, sh-Scb, sh-PITX2 #1, or sh-PITX2 #2 under SD condition, and those treated with ML-SI3 (10 μmol·L^{-1}) or ML-SA1 (20 μmol·L^{-1}). Scale bars: 10 μm. Student's t-test compared the difference in **A**. Data are shown as mean ± s.e.m. (error bars); *, P<0.05; **, P<0.01.

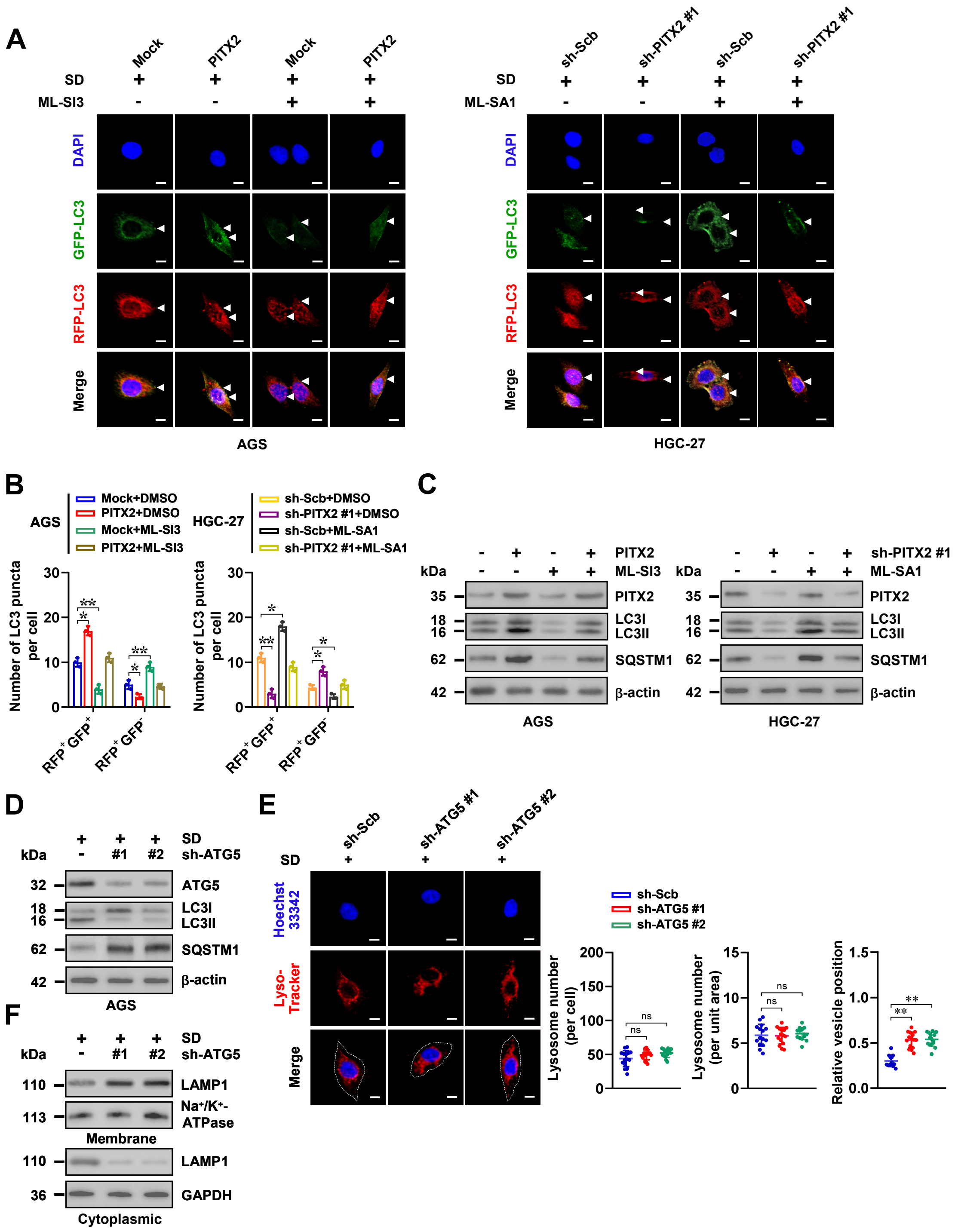

Figure S3. Cross-talk of *PITX2*-mediated lysosomal exocytosis with autophagy. A and B) Representative images (A) and quantification (B) of autophagic flux reporters RFP- and GFP-LC3 (arrowheads) in AGS and HGC-27 cells stably transfected with empty vector (mock), *PITX2*, scramble shRNA (sh-Scb), or sh-PITX2 #1 under SD condition, and those treated with ML-SI3 (10 μmol·L^{-1}) or ML-SA1 (20 μmol·L^{-1}). Scale bars: 10 μm. **C)** Western blot assay indicating the levels of PITX2, LC3, and SQSTM1 in AGS and HGC-27 cells stably transfected with mock, *PITX2*, sh-Scb, or sh-PITX2 #1 under SD condition, and those treated with ML-SI3 (10 μmol·L^{-1}) or ML-SA1 (20 μmol·L^{-1}). **D)** Western blot assay indicating the levels of ATG5, LC3, and SQSTM1 in AGS cells stably transfected with sh-Scb, sh-ATG5 #1, or sh-ATG5 #2 under SD condition. **E)** Representative images (left panel) and quantification (right panels) of Lyso-Tracker Red staining showing the number and anterograde transport distance of lysosomes in AGS cells stably transfected with sh-Scb, sh-ATG5 #1, or sh-ATG5 #2 under SD condition. **F)** Western blot assay indicating the plasma membrane or cytoplasmic expression of LAMP1 in AGS cells stably transfected with sh-Scb, sh-ATG5 #1, or sh-ATG5 #2 under SD condition. One-way ANOVA compared the difference in **B**. Data are shown as mean ± s.e.m. (error bars); *, P<0.05; **, P<0.01.

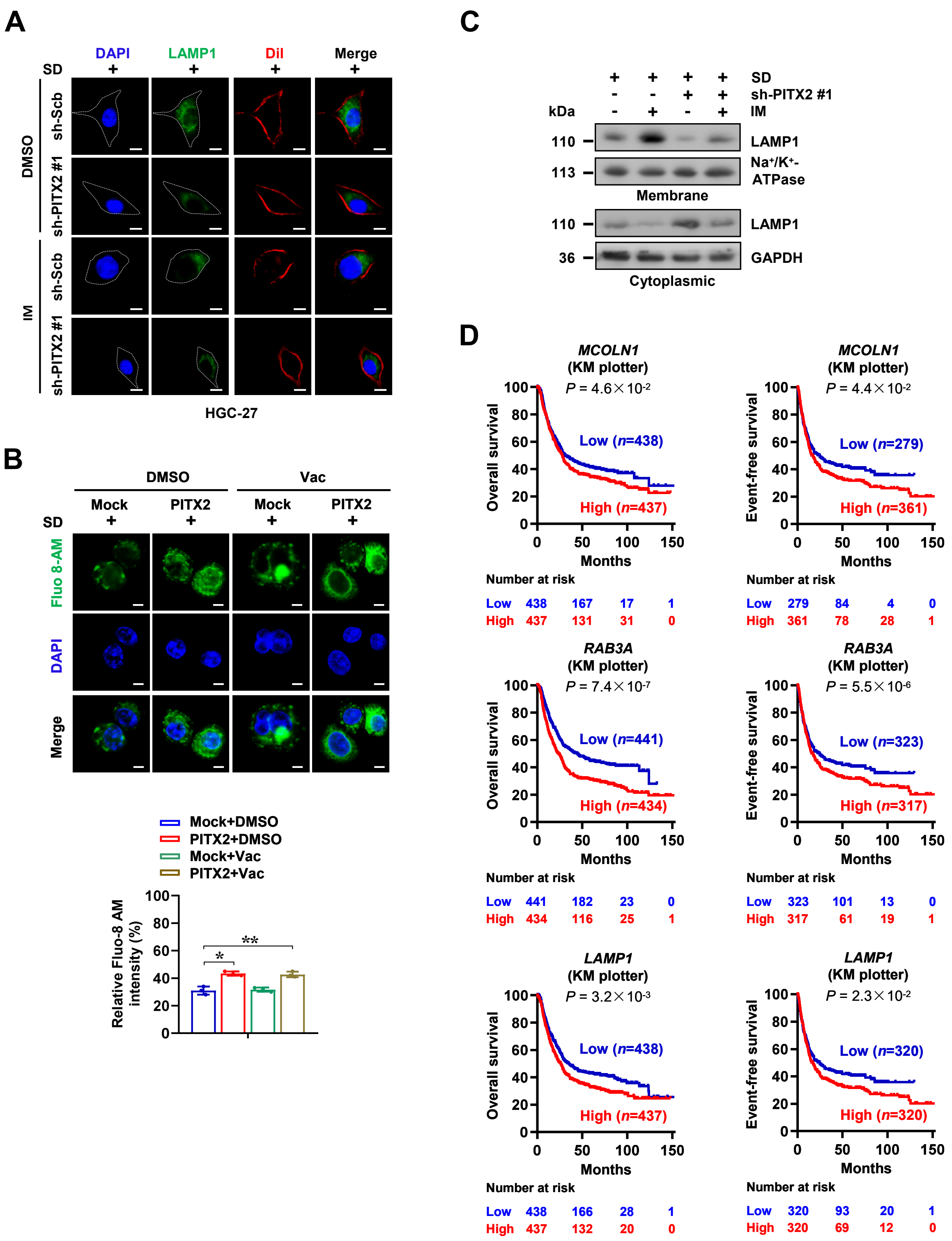

Figure S4. Knockdown of *PITX2* inhibits Ca^{2+}-dependent lysosomal exocytosis of gastric cancer. A) Representative images of LAMP1 and Dil staining in HGC-27 cells stably transfected with scramble shRNA (sh-Scb) or sh-PITX2 #1 under SD condition, and those treated with ionomycin (IM, 10 μmol·L^{-1}). Scale bars: 10 μm. **B**) Representative images (upper panel) and quantification (lower panel) of Fluo 8-AM staining in AGS cells stably transfected with empty vector (mock) or *PITX2* under SD condition, and those treated with DMSO or vacuolin-1 (Vac, 1.0 μmol·L^{-1}), with nucleus staining by DAPI (n=3). Scale bars: 10 μm. **C**) Western blot assay indicating the plasma membrane or cytoplasmic expression of LAMP1 in HGC-27 cells stably transfected with sh-Scb or sh-PITX2 #1 under SD condition, and treated with IM (10 μmol·L^{-1}). **D**) Kaplan-Meier curves indicating overall and event-free survival of gastric cancer cases with low or high levels of *MCOLN1* (cutoff values =7.79 and 7.46), *RAB3A* (cutoff values = 6.13 and 6.00), or *LAMP1* (cutoff values = 9.41 and 12.16). One-way ANOVA compared the difference in **B**. Log-rank test for survival comparison in **D**. Data are shown as mean ± s.e.m. (error bars); *, P<0.05; **, P<0.01.

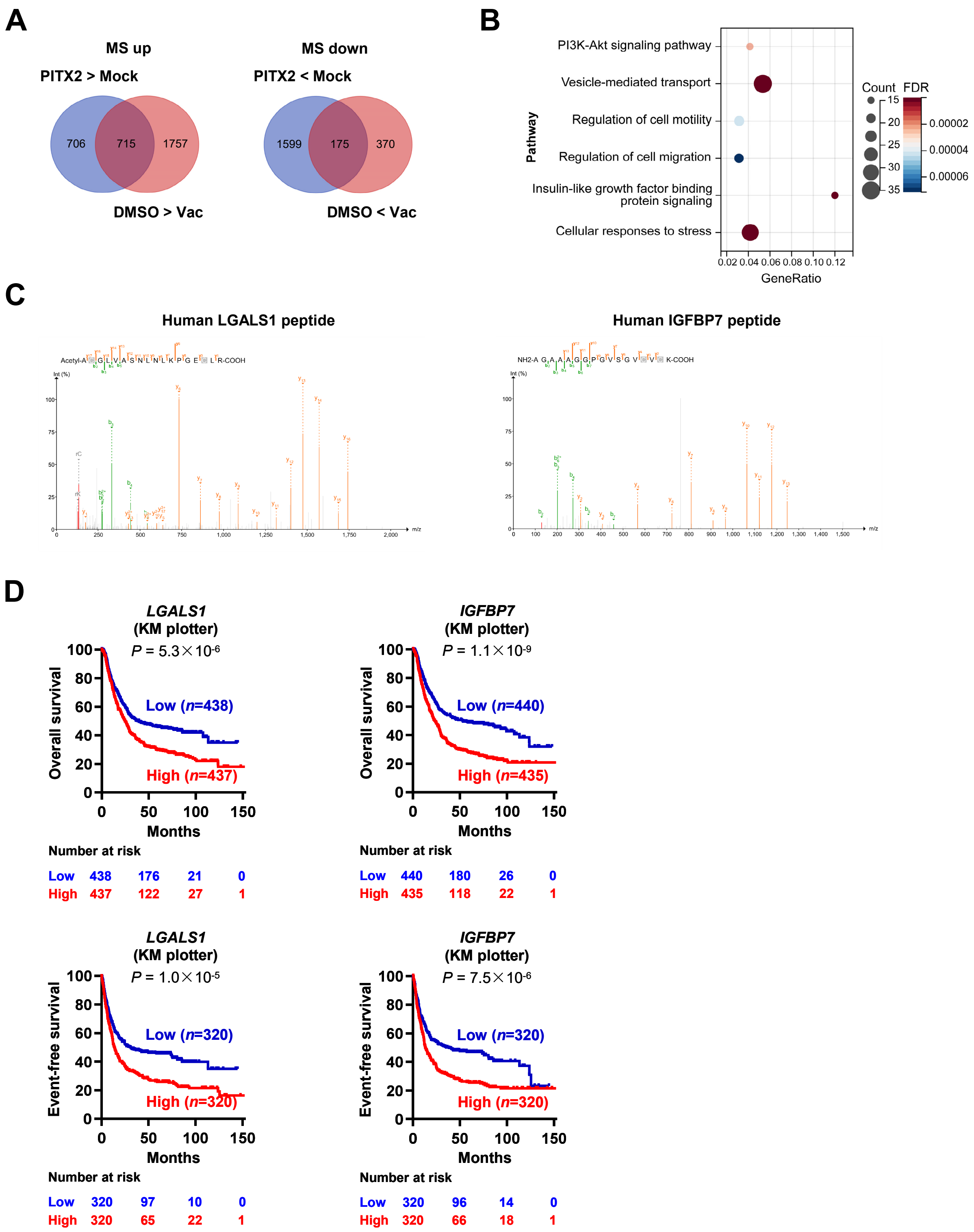

Figure S5. LGALS1 and IGFBP7 are lysosomal exocytosis-secreted proteins associated with poor outcome of gastric cancer. **A**) Venn diagram showing the up-regulated and down-regulated proteins in mass spectrometry (MS) assay of culture medium from AGS cells with stable *PITX2* over-expression or vacuoloin-1 (Vac, 1.0 μmol·L^{-1}) treatment under SD condition. **B**) Bubble graph indicating the involvement of 342 lysosomal exocytosis-secreted SASP proteins in biological pathways. **C**) Peptides of LGALS1 or IGFBP7 in MS assay of culture medium from AGS cells. **D**) Kaplan-Meier curves indicating overall and event-free survival of gastric cancer cases with low or high levels of *LGALS1* (cutoff values = 12.81 and 12.68) or *IGFBP7* (cutoff values = 6.17 and 5.70). Log-rank test for survival comparison in **D**.

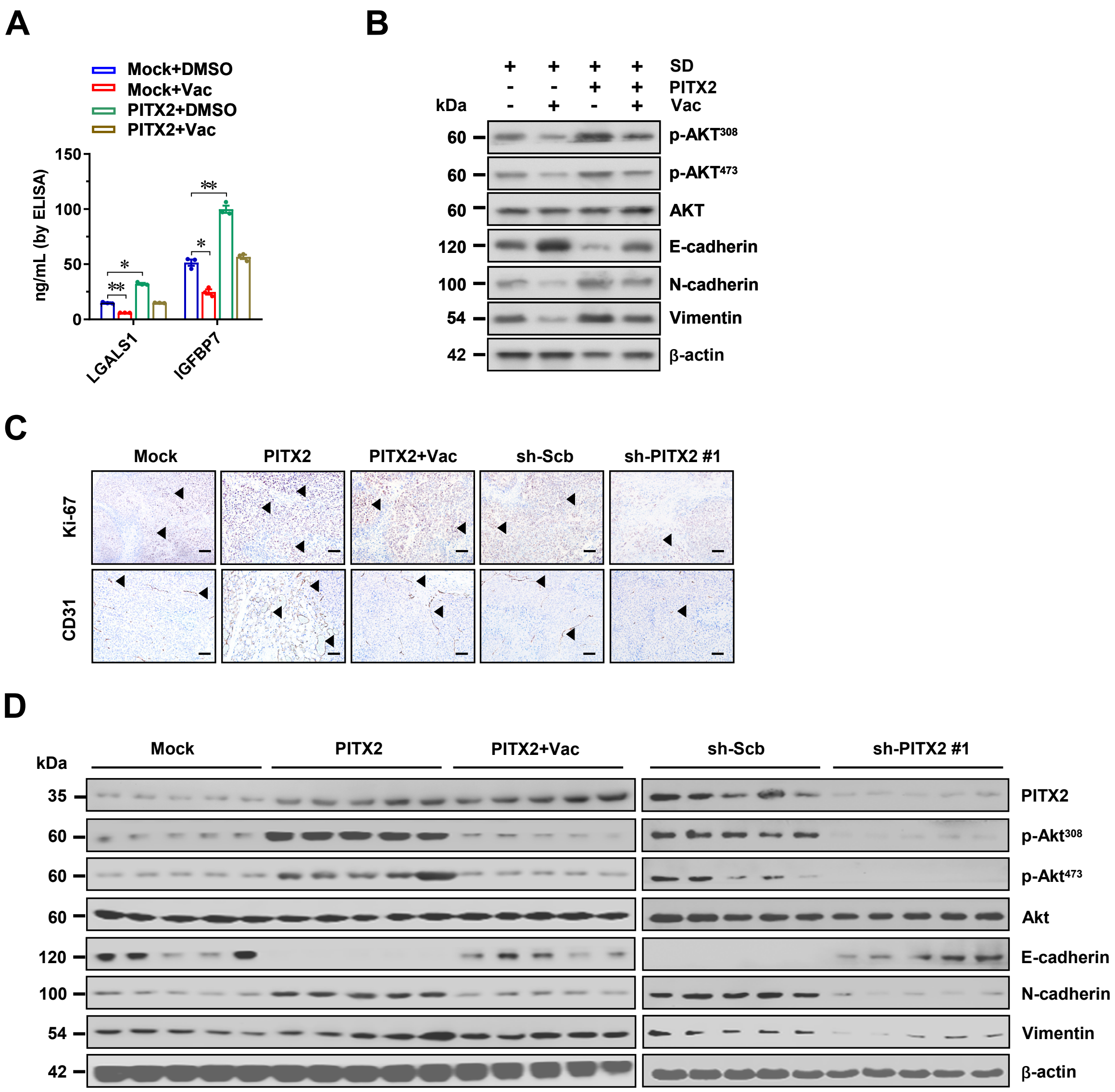

Figure S6. ***PITX2* exerts oncogenic functions via inducing lysosomal exocytosis. A**) ELISA showing the levels of LGALS1 and IGFBP7 within culture medium of senescent AGS cells stably transfected with empty vector (mock) or *PITX2*, and those treated with vacuolin-1 (Vac, 1.0 μmol·L^{-1}, n=3). **B**) Western blot assay indicating the levels of p-AKT^{308}, p-AKT^{473}, E-cadherin, N-cadherin, or vimention in AGS cells with stably transfected with mock or *PITX2* under SD condition, and those treated with Vac (1.0 μmol·L^{-1}). **C**) Representative images of Ki-67 or CD31 expression (arrowheads) in xenograft tumors formed by subcutaneous injection of AGS or HGC-27 cells in nude mice (n=5 for each group) that subsequently treated with intravenous injection of culture medium collected from senescent cells stably transfected with mock, *PITX2*, scramble shRNA (sh-Scb), sh-PITX2 #1, with or without Vac (1.0 μmol·L^{-1}) treatment. **D**) Western blot assay showing the expression of p-AKT^{308}, p-AKT^{473}, E-cadherin, N-cadherin, or vimentin in xenograft tumors formed by subcutaneous injection of AGS or HGC-27 cells in nude mice (n=5 for each group) that subsequently treated with intravenous injection of culture medium collected from senescent cells stably transfected with mock, PITX2, sh-Scb, or sh-PITX2 #1, with or without Vac (1.0 μmol·L^{-1}) treatment. One-way ANOVA compared the difference in **A**. Data are shown as mean ± s.e.m. (error bars); *, P<0.05; **, P<0.01.

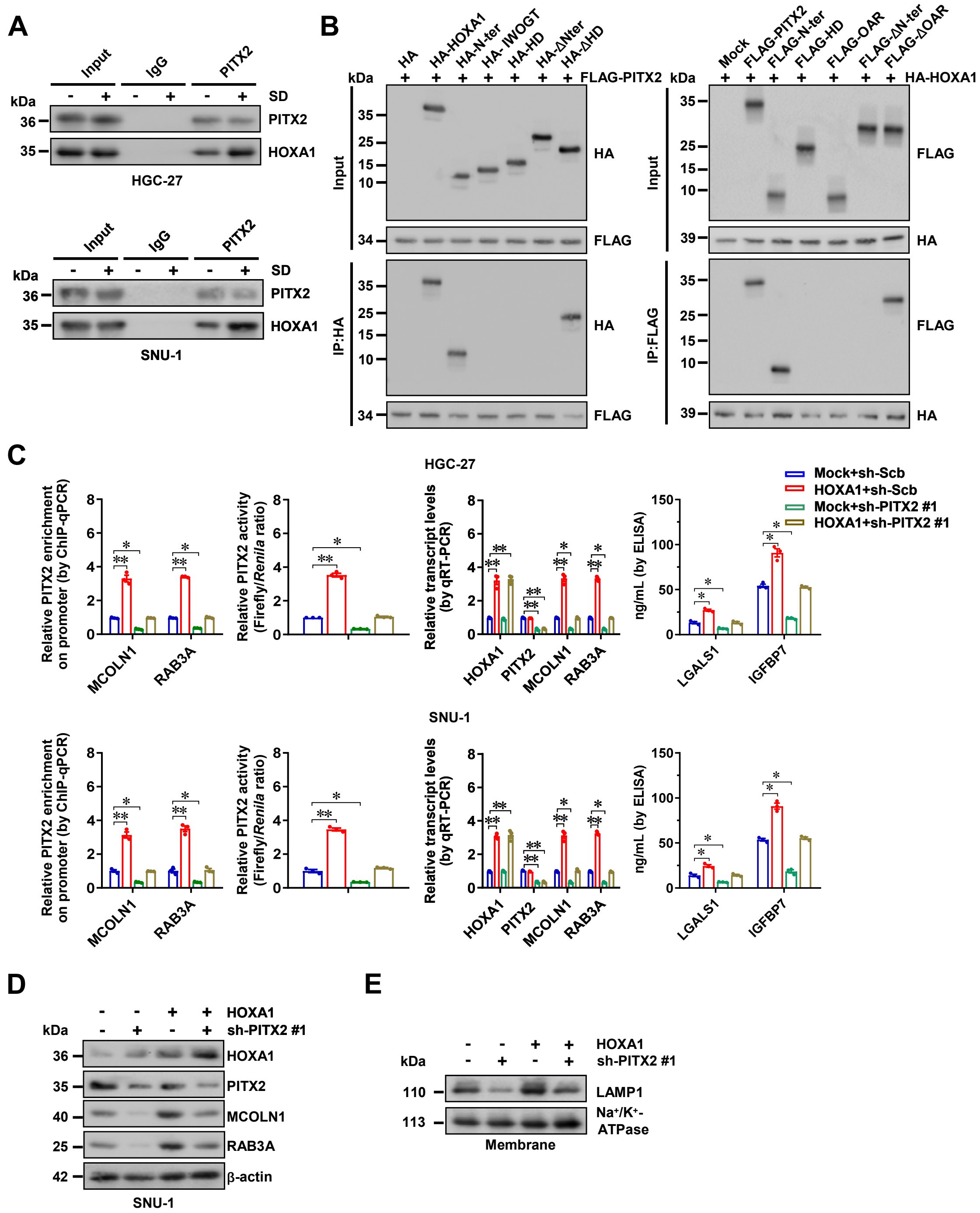

Figure S7. HOXA1 cooperates with PITX2 to induce lysosomal exocytosis in gastric cancer cells. A) Co-IP and western blot assays indicating the interaction between PITX2 and HOXA1 in HGC-27 and SNU1 cells under complete medium (CM) or serum deprivation (SD) condition. **B)** Co-IP and western blot assays indicating the interaction between HA-tagged HOXA1 and FLAG-tagged PITX2 truncation proteins in AGS cells transfected with their corresponding constructs. **C)** ChIP-qPCR (normalized to input, *n*=3), dual-luciferase reporter (*n*=3), real-time qRT-PCR (normalized to *β-actin*, *n*=3), and ELISA assays showing the PITX2 enrichment on target gene promoter region, PITX2 activity, transcript levels of *MCOLN1* or *RAB3A*, and LGALS1 or IGFBP7 secretion of HGC-27 and SNU-1 cells stably transfected with mock or *HOXA1*, and those co-transfected with scramble shRNA (sh-Scb) or sh-PITX2 #1. **D)** Western blot assay showing the levels of target genes MCOLN1 and RAB3A in SNU-1 cells stably transfected with mock or *HOXA1*, and those co-transfected with sh-Scb or sh-PITX2 #1. **E)** Western blot assay indicating the plasma membrane expression of LAMP1 in HGC-27 cells stably transfected with mock or *HOXA1*, and those co-transfected with sh-Scb or sh-PITX2 #1. One-way ANOVA compared the difference in **C**. Data are shown as mean ± s.e.m. (error bars); *, $P<0.05$; **, $P<0.01$.

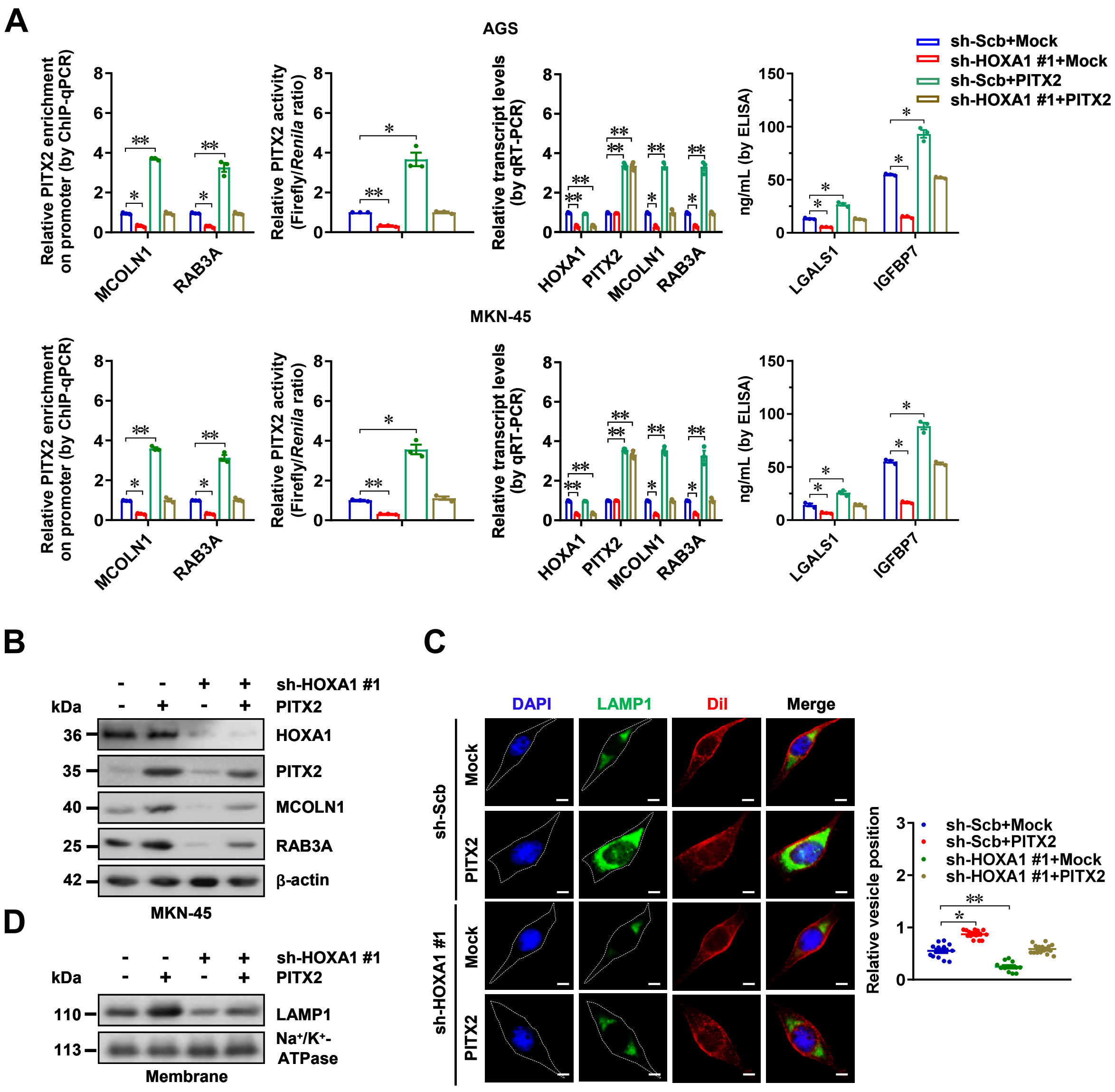

Figure S8. Knockdown of *HOXA1* attenuates the roles of *PITX2* in promoting lysosomal exocytosis of gastric cancer cells. A) ChIP-qPCR (normalized to input, *n*=3), dual-luciferase reporter (*n*=3), real-time qRT-PCR (normalized to *β-actin*, *n*=3), and ELISA (*n*=3) assays showing the PITX2 enrichment on target gene promoter region, PITX2 activity, transcript levels of *MCOLN1* or *RAB3A*, and LGALS1 or IGFBP7 secretion of AGS and MKN-45 cells stably transfected with empty vector (mock) or *PITX2*, and those co-transfected with scramble shRNA (sh-Scb) or sh-HOXA1 #1. **B**) Western blot assay showing the levels of target genes MCOLN1 and RAB3A in MKN-45 cells stably transfected with mock or *PITX2*, and those co-transfected with sh-Scb or sh-HOXA1 #1. **C**) Representative images (left panel) and quantification (right panel) of LAMP1 and Dil staining in AGS cells stably transfected with mock or *PITX2*, and those co-transfected with sh-Scb or sh-HOXA1 #1 (*n*=15). Scale bars: 10 µm. **D**) Western blot assay indicating the plasma membrane expression of LAMP1 in AGS cells stably transfected with mock or *PITX2*, and those co-transfected with sh-Scb or sh-HOXA1 #1. One-way ANOVA compared the difference in **A** and **C**. Data are shown as mean ± s.e.m. (error bars); *, *P*<0.05; **, *P*<0.01.

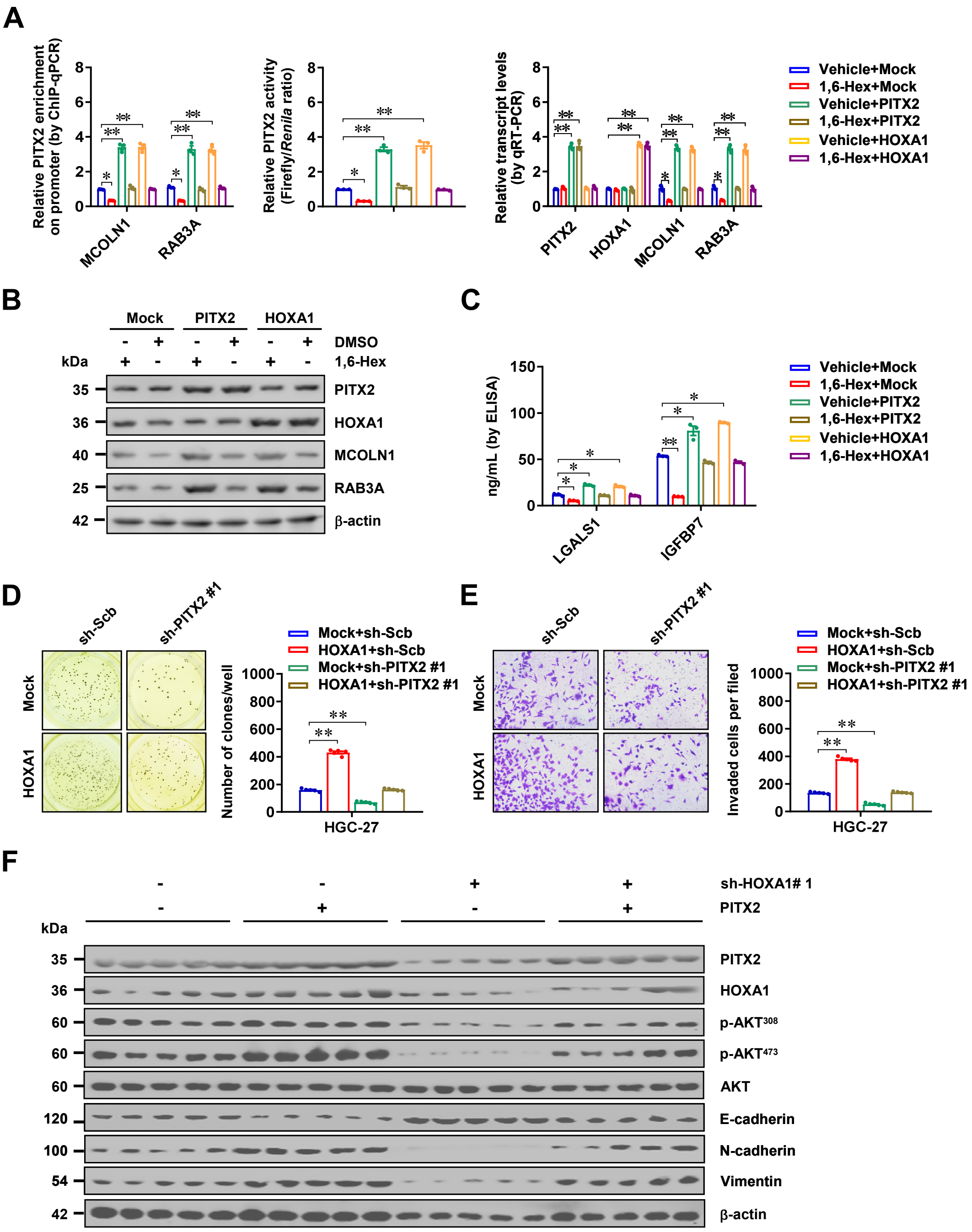

Figure S9. HOXA1/PITX2 condensates promote lysosomal exocytosis and gastric cancer progression. **A**-**C**) ChIP-qPCR (A, normalized to input, *n*=3), dual-luciferase reporter (A, *n*=3), real-time qRT-PCR (A, normalized to *β-actin*, *n*=3), western blot (B), and ELISA (C, *n*=3) assays showing the PITX2 enrichment on target gene promoter region, PITX2 activity, *MCOLN1* or *RAB3A* expression, and LGALS1 or IGFBP7 secretion of AGS cells stably transfected with empty vector (mock), *PITX2*, or *HOXA1*, and those treated with DMSO or 1.5% 1,6-Hex. **D** and **E**) Representative images (left panel) and quantification (right panel) of soft agar (D) and matrigel invasion (E) assays indicating anchorage-independent growth and invasion capability of HGC-27 cells treated by culture medium form senescent cells stably transfected with mock or *HOXA1*, and those co-transfected with scramble shRNA (sh-Scb) or sh-PITX2 #1 (*n*=5). **F**) Western blot assay showing the expression of p-AKT^{308}, p-AKT^{473}, E-cadherin, N-cadherin, or Vimention in tumor xenografts formed by subcutaneous injection of MKN-45 cells in nude mice that subsequently treated with intravenous injection of culture medium collected from senescent cells stably transfected with sh-Scb or sh-HOXA1 #1, and those co-transfected with mock or *PITX2* (*n*=5 for each group). One-way ANOVA compared the difference in **A** and **C**-**E**. Data are shown as mean ± s.e.m. (error bars); *, P<0.05; **, P<0.01.

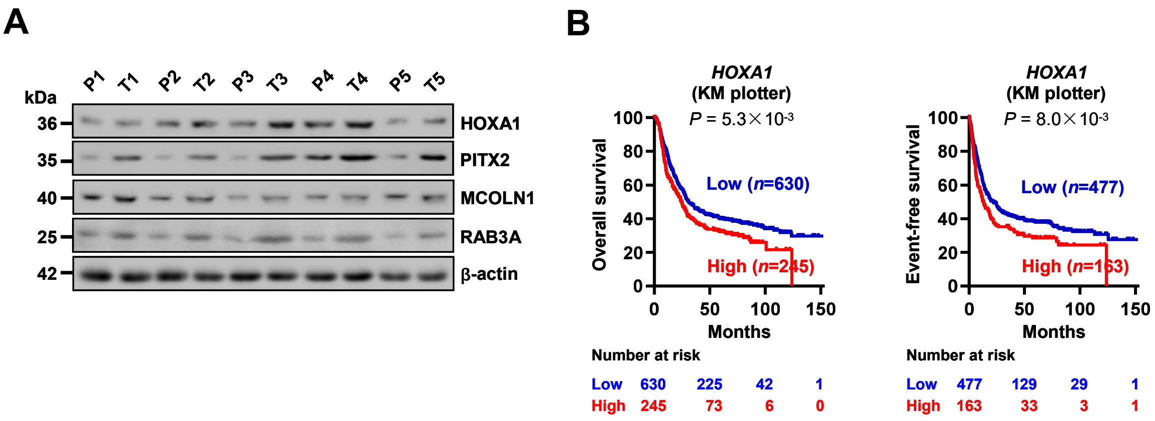

Figure S10. Expression profiles of *HOXA1*, *PIXT2* and target genes in gastric cancer progression. A) Western blot assay showing the expression of HOXA1, PITX2, MCOLN1, and RAB3A in tumor (T) and para-tumoral (P) tissues of gastric cancer. **B**) Kaplan-Meier curves indicating overall and event-free survival of gastric cancer cases with low or high levels of *HOXA1* (cutoff values = 7.28 and 7.46). Log-rank test for survival comparison in **B**.

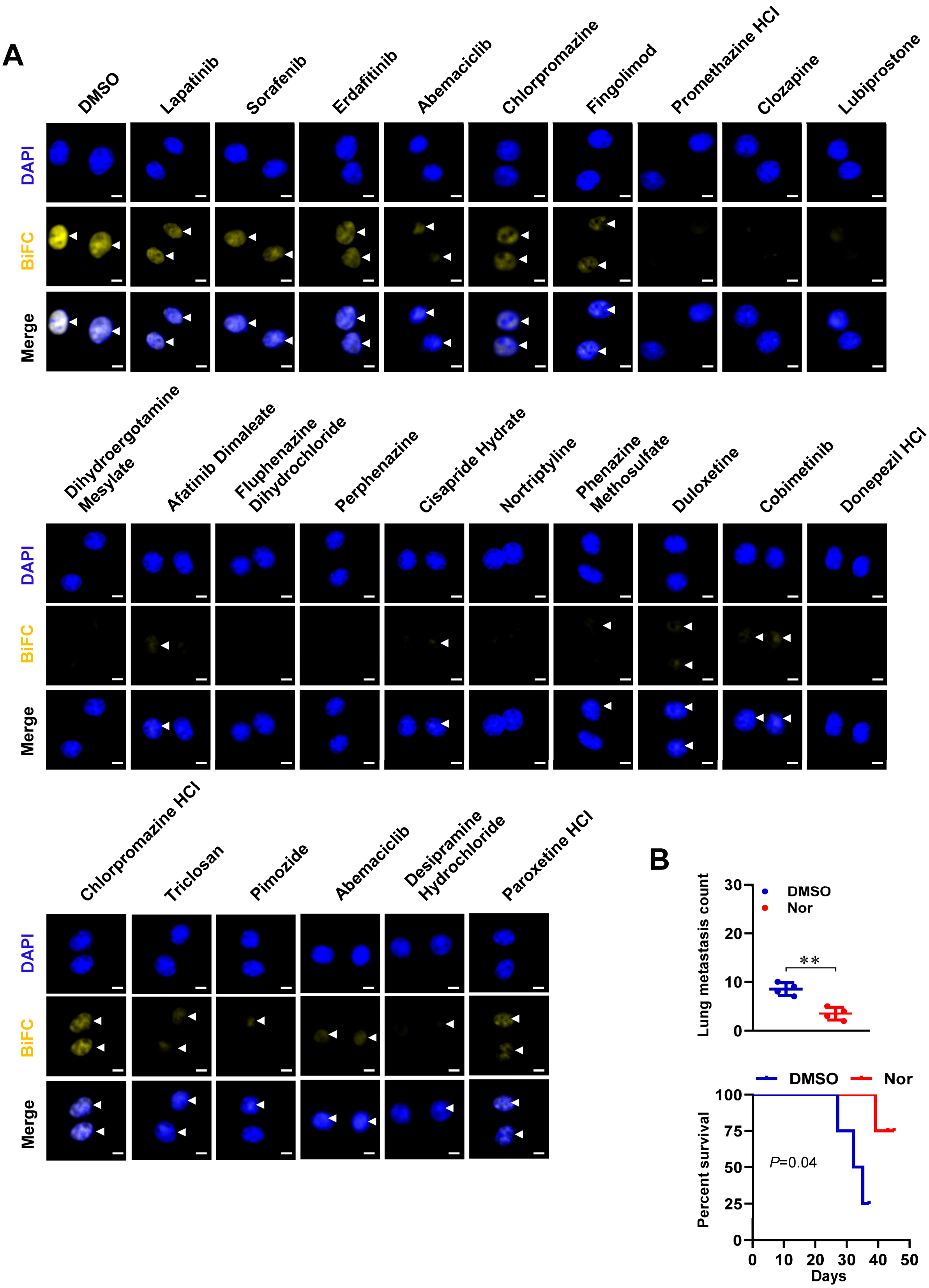

Figure S11. Screening of inhibitors repressing HOXA1-PITX2 interaction. A) Representative images and quantification of BiFC assay showing the effects of chemicals on physical interaction (arrowheads) of HOXA1 and PITX2 in AGS cells transfected with pBiFC-VN173-HOXA1 and pBiFC-VC155-PITX2. Scale bars: 10 μm. **B**) Kaplan-Meier curves of nude mice (*n*=4 for each group) receiving vein tail injection of HGC-27 cells that subsequently treated with intravenous injection of Nor (50 mg·kg^{-1}). Student's *t*-test compared the difference in **B**. Log-rank test for survival comparison in **B**. Data are shown as mean ± s.e.m. (error bars); **, *P*<0.01.

Table S1 Transcription factors of differentially expressed lysosomal exocytosis genes

Lysosomal exocytosis genes	Transcription factors								
DOC2A	PITX2	E2F1	FOXJ1	HOXD9	MAX	NKX3-2	PLAU	SOX17	TEAD2
MCOLN1	AHR	E2F6	FOXL1	IKZF1	MEF2A	NOBOX	POU1F1	SOX2	TFAP2A
RAB3A	AP1	EBF1	GABPA	IRF2	MIR133B	NR1H2	POU2F2	SOX5	TFAP2D
SNAPIN	APEX1	EGR1	GATA1	IRF8	MTF1	NR1H3	POU5F1	SPI1	THRB
SPHK2	ARID3A	ELF5	GATA2	JUN	MYB	NR2E3	PPARG	SPIB	TP53
SYNGR1	ARNT	ELK4	GATA6	JUND	MYC	NR3C1	PRRX2	SPZ1	TP63
SYT11	ATF4	EN1	GFI1	KLF11	MYCN	NR5A1	RBPJ	SREBF1	UBTF
VAMP7	BRCA1	ESRRB	GLI2	KLF13	MYOG	NR5A2	RELA	SREBF2	USF1
VTI1B	CACYBP	ETS1	HIF1A	KLF4	MZF1	NRF1	RUNX1	SRF	USF2
	CBEPB	EVI1	HINFP	KLF5	NFATC3	PAX2	RUNX2	STAT3	WT1
	CEBPB	FOS	HNF1A	LEF1	NFE2	PAX4	RXRA	TBP	YY1
	CEBPD	FOXA1	HNF1B	LHX3	NFIC	PAX5	SMAD4	TCF4	ZFHX3
	CREB1	FOXA2	HNF4A	LTF	NFKB1	PDX1	SMARCA2	TCFCP2L1	ZFX
	CRX	FOXF2	HOXA5	MAPK14	NKX2-5	PITX1	SOX10	TEAD1	ZNF148

Table S2 Mass spectrometry analysis of PITX2-upregulated SASP proteins via lysosomal exocytosis

AAMDC	DPYSL3	MANBA	RNPEP
ACAT2	DSTN	MAPRE1	RPS12
ACBD3	DTD1	MIF	RPS21
ACOT7	DUSP3	MKI67	RRBP1
ACY1	ECH1	MMP2	RRM2
AHNAK	EIF4A2	MTPN	RRM2B
AHNAK2	EIF4EBP1	MUC13	RSU1
AIP	EIF4G3	MVD	S100A10
AK1	EIF4H	MYLK	S100A16
ALDH16A1	EPS8L2	NAPA	SART1
ALDOA	FDPS	NEDD8	SEC23A
ANXA7	FH	NES	SERPINB1
AP2A1	FHL2	NIF3L1	SERPINB6
APEX1	FKBP1A	NIT2	SF3A1
ARHGAP1	FKBP3	NLN	SF3B1
ARHGDIA	FUBP1	NONO	SIN3A
ARPC1B	G3BP1	NOP2	SLC9A3R2
ARPC2	GAA	NUFIP2	SNRPC
ASL	GALE	OLA1	SORBS3
ATOX1	GALNS	OPTN	SORD
B2M	GBE1	OTUB1	SPIN1
BAG3	GDI1	PAFAH1B2	SRI
BID	GDI2	PAICS	SRP9
BPNT1	GGCT	PARVA	SRSF1
BSG	GGH	PCBD1	SRSF2
CAB39	GLO1	PCBP1	SRSF7
CALML5	GMFB	PCMT1	STAT1
CAP1	GNB1	PDCD5	SUMO1
CAPN1	GNB2	PDLIM1	SUPT5H
CAPNS1	GRB2	PDLIM4	TAGLN2
CASP14	GSTO1	PDLIM5	TAX1BP3
CASP3	GSTP1	PDXK	TBC1D4
CD2AP	GUK1	PEA15	TBCA
CD46	GYG1	PFDN1	TBCB
CDV3	HADH	PGAM1	TES
CFL1	HINT1	PGD	TFG
CHMP1A	HLA-B	PGM1	TOLLIP
CMPK1	HNRNPA1	PGM2	TOM1
CNN2	HNRNPL	PGM2L1	TPT1
CNN3	HSD17B4	PICALM	TWF1
CORO1B	HSPG2	PLIN3	TWF2
COX5A	IGFBP7	PLS3	TXN
COX5B	IMPA1	PLXNB2	TXNDC5
COX6B1	ISOC1	PNP	UBAP2L
CPOX	ITGA2	PPA1	UBE2K
CRIP1	ITGB4	PPIA	UBE2V1
CRK	ITM2B	PPIH	UCHL3
CROCC	ITSN2	PPP1R14B	UFC1
CRYZ	JUP	PRDX1	UFM1
CSRP1	LAMC2	PRDX3	UGP2
CSTB	LAMP2	PRDX6	URM1
CTSC	LAMTOR1	PRRC2C	UROD
CYCS	LAMTOR3	PSME2	UTRN
DBI	LAMTOR5	PTBP1	VAPA
DBNL	LARP7	RAB1A	VASP
DCTPP1	LDHB	RAB2A	VAT1
DDRGK1	LDLR	RAB5C	VCL
DIDO1	LGALS1	RAB6A	WDHD1
DNAJB1	LGALS3	RAD23A	WDR1
DNAJC8	LPP	RAD23B	XPNPEP1
DPP7	LTA4H	RBM22	YEATS4
DPY30	MAN2B1	RBM7	ZYX

Table S3 Mass spectrometry analysis of PITX2-reduced SASP proteins via lysosomal exocytosis

A2M	EEF1B2	PLOD3	VASN
AGRN	ENO3	PPIL4	WNT5A
ANP32B	FBLN1	PRKCSH	YWHAE
ARHGEF18	FBN1	PRKRA	YWHAG
ASCC3	FN1	PSAP	
ATRN	FSTL1	PSMA3	
AXL	GLUD1	PXDN	
BASP1	GNS	RANBP1	
BLMH	GOLGA1	RCN1	
BMS1	GPC1	RDX	
BTD	GRN	RPL10A	
C3	HBB	RPL13	
CALR	HDGF	RPL23	
CALU	HSPA13	SAFB	
CD44	HSPA5	SDC4	
CD63	HSPA6	SDF4	
CDA	HSPA9	SERPINE1	
CDH13	IGFBP2	SLK	
CDH2	LAMA4	SPARC	
CNPY2	LAMA5	SPOCK1	
COL4A2	LAMC1	SPTAN1	
COL5A1	LMNA	SPTBN1	
COL5A2	LMNB2	SUB1	
COL7A1	LOXL2	SUMF2	
CTSB	NRP1	TIMP2	
CTSL	NUCB2	TNC	
CTSZ	P4HB	TPM1	
CXADR	PDAP1	TPM2	
DBN1	PDIA3	TPM3	
DDX21	PLOD2	TPM4	

Table S4 Comprehensive analysis of PITX2-interacting proteins

HURI	InBioMap	BioGRID	IID			
AES	CREBBP	AES	AHCYL1	HNRNPK	MAPK8	RGS11
ANKRD10	CTNNB1	AHCYL1	AHCYL2	HNRNPL	MEF2A	RGS12
ATN1	EP300	AHCYL2	AKAP10	HNRNPU	MEOX2	RGS16
CTNNB1	FOXC1	APEX1	ALX1	HOXA1	MIPOL1	RGS17
CYSRT1	HDAC1	ATN1	ALX3	HOXA10	MNX1	RGS18
DAZAP2	HERC5	CIC	ALX4	HOXA11	MSX2	RGS19
DDIT4L	HNRNPK	CTNNB1	ARRB1	HOXA13	MYBL1	RGS2
DYNLT1	HNRNPU	CYSRT1	ARRB2	HOXA2	MYC	RGS3
HEY1	HOXA1	DAZAP2	AVP	HOXA3	MYOC	RGS4
HOXA1	KAT5	DYNLT1	BARHL2	HOXA4	NCK2	RGS5
KRT34	LDB1	FOXC2	BARX1	HOXA5	NCL	RGS6
KRTAP15-1	LEF1	HDAC1	BARX2	HOXA6	NFE2L2	RGS7
KRTAP19-5	MEF2A	HERC5	CDC37	HOXA7	NFKB2	RGS8
KRTAP19-7	NCL	HEY1	CDC73	HOXA9	NKX2-1	RGS9
KRTAP3-3	NFE2L2	HOXA1	CDK1	HOXB1	NKX2-2	SATB2
KRTAP6-1	PDLIM1	IER2	CDK2	HOXB2	NKX2-3	SIX3
KRTAP7-1	PDLIM4	KRT34	CDK3	HOXB3	NKX2-5	SMAD3
OIP5	POU1F1	KRTAP15-1	CDK7	HOXB4	NKX3-1	SMO
PFDN5	SMAD3	KRTAP19-5	CDX1	HOXB5	NKX3-2	SNX13
POU2AF1	TRIM25	KRTAP19-7	CDX4	HOXB6	NKX6-2	SP2
PSMB4	WDR5	KRTAP3-3	CFC1	HOXB7	NOTCH1	SP8
RBPMS	YAP1	KRTAP6-1	CTCF	HOXB8	NPM1	SRSF9
SMUG1	YBX1	KRTAP7-1	CTNNB1	HOXB9	NR3C1	STAU1
TBX22		LEF1	CUL1	HOXC10	NUP62	TFAP2A
TEX37		LRRC46	CYP1B1	HOXC11	OSTM1	THEG
TINAGL1		MSX2	DDX1	HOXC13	OTP	TLX1
TRAF1		N	DDX58	HOXC4	OTX1	TLX2
TRIB3		OIP5	DLX1	HOXC5	PAX3	TLX3
TRIM25		PFDN5	DLX2	HOXC6	PAX4	TOP1
UFSP1		Pou1f1	DLX5	HOXC8	PAX7	TP53
VGLL3		POU2AF1	EMX2	HOXD10	PAX8	TRIM25
WWOX		PSMB4	EN1	HOXD11	PBX2	UNCX
ZNF34		RBPJ	EN2	HOXD13	PBX3	VAX2
		RBPMS	EP300	HOXD3	PCBP2	VSX1
		SMAD3	ESR1	HOXD4	PDLIM1	VSX2
		TEX37	EVX1	HOXD8	PDX1	WDR5
		TINAGL1	EVX2	HOXD9	PLOD1	WT1
		TRAF1	FOXC1	HSP90AB1	PLOD2	YAP1
		TRIB3	FOXC2	HSPA8	POU1F1	YBX1
		TRIM25	FOXE3	IER2	POU2F1	ZEB2
		UFSP1	FOXF1	IKBKB	POU3F1	ZIC3
		VGLL3	FOXF2	IRX5	POU3F3	ZNHIT3
		WDR5	FOXH1	IRX6	POU4F1	
		WNT2	FOXJ1	JUP	POU4F3	
		WNT2B	GBX2	KAT2B	PPM1G	
		WNT5A	GIPC1	KAT5	PPP1CB	
		WNT6	GPHA2	KIF3B	PPP2R2B	
		WNT9A	GPR158	KLF10	PRKACB	
		WWOX	GPR17	KLF13	PROP1	
		ZNF34	GRB2	KLF14	PRRX1	
		ZNHIT3	GSC	KLF16	PRRX2	
			GSK3B	KLF9	PTBP1	
			GSX2	LDB1	PURA	
			GTF2F1	LEF1	RAX	
			HABP4	LHX1	RBBP6	
			HDAC1	LHX8	RBMX	
			HERC5	LMX1B	RBPJ	
			HHEX	LRRC46	RELA	
			HIF1A	MAP3K14	RFC2	
			HMX3	MAPK1	RGS1	
			HNRNPD	MAPK10	RGS10	

Table S5 Primer sets used for RT-PCR, qPCR, and ChIP

Primer set	Primers	Sequence	Product size (bp)	Application
PITX2	Forward	5’-CGCGGATCCATGGAGACCAACTGCCGCAAAC-3'	813	RT-PCR
(Variant 1)	Reverse	5’-GCGCACCGGTCACGGGCCGGTCCACTGCATAC-3'		
PITX2	Forward	5’-CGCGGATCCATGAACTGCATGAAAGGCCCGC-3'	972	RT-PCR
(Variant 3)	Reverse	5’-GCGCACCGGTCACGGGCCGGTCCACTGCATAC-3'		
PITX2	Forward	5'-CAAAGGCGGCAGCGGACTCACT-3’	292	qPCR
(Variant 1)	Reverse	5'-TGGCGGCCCAGTTGTTGTAGGA-3’		
PITX2	Forward	5'-TGAAAGGCCCGCTTCACT-3’	268	qPCR
(Variant 3)	Reverse	5'-CTTTGCCGCTTCTTCTTA-3’		
HOXA1	Forward	5'-ACTCCTTGTCCCCTCTCCACGC-3’	212	qPCR
	Reverse	5'-GAACTCCTTCTCCAGTTCCGTG-3’		
RAB3A	Forward	5'-CAGGTGCTGCTGGTAGGAAA-3’	102	qPCR
	Reverse	5'-AAAGAACTCGAACCCAAGGTG-3’		
MCOLN1	Forward	5'-TCCGCTACCTGACCTTCTTCCA-3'	222	qPCR
	Reverse	5'-TCACAAACATGTCGTCCCCATT-3'		
DOC2A	Forward	5'-CACAGATGACGACATCACGCACAA-3'	191	qPCR
	Reverse	5'-GCGCCGCTGACATGGAAGAG-3'		
SNAPIN	Forward	5'-AATTGACAACCTAGCCACAGAAC-3'	159	qPCR
	Reverse	5'-TTTAGCCGTCTCAGTCGTTCC-3'		
SYNGR1	Forward	5'-GCCTTCCTCACCTGCCTGCTGT-3'	198	qPCR
	Reverse	5'-CCCTTCGTTCAGTGGGTTGTCC-3'		
VAMP7	Forward	5'-GGTAGCTCAGCGAGGAGAAAG-3'	207	qPCR
	Reverse	5'-GGCCATGTAAATCCACCACAG-3'		
VTI1B	Forward	5'-AAGGACCTTGCTAAACTCCA-3'	241	qPCR
	Reverse	5'-CTTCTATGATTTCTGAGCCAAT-3'		
ACTB	Forward	5'-TGCCCATCTACGAGGGGTATG-3'	156	qPCR
	Reverse	5'-TCTCCTTAATGTCACGCACGATTT-3'		
RAB3A	Forward	5'-CGTGACGTCCTGCAAAGGGA-3'	298	ChIP
	Reverse	5'-GGCTACTTCGCCCGTCGCTC-3'		
MCOLN1	Forward	5'-GGAATGTTGGAAGACTCTGGGC-3'	288	ChIP
	Reverse	5'-ATGGGCTGACTCTGAGTTTGGC-3'		

PITX2, Paired like homeodomain 2; HOXA1, Homeobox A1; RAB3A, RAS-related protein Rab-3A; MCOLN1, Mucolipin 1; DOC2A, double C2 domain alpha; SNAPIN, SNAP associated protein; SYNGR1, synaptogyrin 1; VAMP7, vesicle associated membrane protein 7; VTI1B, vesicle transport through interaction with t-SNAREs 1B; ACTB, beta-actin; ChIP, chromatin immunoprecipitation.

Table S6 Oligonucleotide sets used for constructs

Oligo Set	Sequences
pGL4.31-PITX2 Luc	5'-CATAATCCCTAATAATCCATAATAATTCCTAATAATTCAA-3' (Sense);<br>5'-AGCTTTGAATTATTAGGAATTATTATGGATTATTAGGGATTATGGTAC-3' (Antisense)
Lenti-CV186-PITX2 (1-816)	5’-CGCGGATCCATGGAGACCAACTGCCGCAAAC-3' (Sense);<br>5’-GCGCACCGGTCACGGGCCGGTCCACTGCATAC-3' (Antisense)
pCMV-3Tag-PITX2 (1-816)	5’-CGCGGATCCATGGAGACCAACTGCCGCAA-3' (Sense);<br>5’-CCGAAGCTTTCACACGGGCCGGTCCACTG-3'' (Antisense)
pCMV-3Tag-PITX2 (1-114)	5’-CGCGGATCCATGGAGACCAACTGCCGCAA-3' (Sense);<br>5’-CCGAAGCTTCCGCTTCTTCTTAGACGGGT-3'' (Antisense)
pCMV-3Tag-PITX2 (115-696)	5’-CGCGGATCCCAAAGGCGGCAGCGGACTCA-3’’ (Sense);<br>5’-CCGAAGCTTGTTACACGTGTCCCTATAAA-3’ (Antisense)
pCMV-3Tag-PITX2 (697-816)	5’-CGCGGATCCTCGAGCCTGGCCAGCCTGAG-3’ (Sense);<br>5’-CCGAAGCTTTCACACGGGCCGGTCCACTG-3’ (Antisense)
pCMV-3Tag-1A-PITX2 (115-816)	5’-CGCGGATCCCAAAGGCGGCAGCGGACTCA-3’ (Sense);<br>5’-CCGAAGCTTTCACACGGGCCGGTCCACTG-3’ (Antisense)
pCMV-3Tag-PITX2 (1-696)	5’-CGCGGATCCATGGAGACCAACTGCCGCAA-3’ (Sense);<br>5’-CCGAAGCTTGTTACACGTGTCCCTATAAA-3’’ (Antisense)
pMAL-c4X-PITX2 (1-816)	5’-CGCGGATCCATGGAGACCAACTGCCGCAA-3' (Sense);<br>5’-CCGAAGCTTTCACACGGGCCGGTCCACTG-3'' (Antisense)
pMAL-c4X-PITX2 (1-114)	5’-CGCGGATCCATGGAGACCAACTGCCGCAA-3' (Sense);<br>5’-CCGAAGCTTCCGCTTCTTCTTAGACGGGT-3'' (Antisense)
pMAL-c4X-PITX2 (115-696)	5’-CGCGGATCCCAAAGGCGGCAGCGGACTCA-3’’ (Sense);<br>5’-CCGAAGCTTGTTACACGTGTCCCTATAAA-3’ (Antisense)
pMAL-c4X-PITX2 (697-816)	5’-CGCGGATCCTCGAGCCTGGCCAGCCTGAG-3’ (Sense);<br>5’-CCGAAGCTTTCACACGGGCCGGTCCACTG-3’ (Antisense)
pMAL-c4X-PITX2 (115-816)	5’-CGCGGATCCCAAAGGCGGCAGCGGACTCA-3’ (Sense);<br>5’-CCGAAGCTTTCACACGGGCCGGTCCACTG-3’ (Antisense)
pMAL-c4X-PITX2 (1-696)	5’-CGCGGATCCATGGAGACCAACTGCCGCAA-3’ (Sense);<br>5’-CCGAAGCTTGTTACACGTGTCCCTATAAA-3' (Antisense)
pCMV-HA-HOXA1 (1-1008)	5’-GGAGAATTCGGATGGACAATGCAAGAATGAA-3' (Sense)<br>5’-CCGCTCGAGTCAGTGGGAGGTAGTCAGAG-3' (Antisense)
pCMV-HA-HOXA1 (1-249)	5’-GGAGAATTCGGATGGACAATGCAAGAATGAA-3'' (Sense)<br>5’-CCGCTCGAGGGAAGTCTGGTAGGTAGCCG-3'' (Antisense)
pCMV-HA-HOXA1 (250-609)	5’-GGAGAATTCGGGGGAACCTGGGGGTGTCCTA-3' (Sense)<br>5’-CCGCTCGAGCTGCGCTGGAGAAGATGTCT-3' (Antisense)
pCMV-HA-HOXA1 (610-1008)	5’-GGAGAATTCGGACTTTTGACTGGATGAAAGT-3' (Sense)<br>5’-CCGCTCGAGTCAGTGGGAGGTAGTCAGAG-3' (Antisense)
pCMV-HA-HOXA1 (250-1008)	5’-GGAGAATTCGGGGGAACCTGGGGGTGTCCTA-3' (Sense)<br>5’-CCGCTCGAGTCAGTGGGAGGTAGTCAGAG-3' (Antisense)
pCMV-HA-HOXA1 (1-609)	5'-GGAGAATTCGGATGGACAATGCAAGAATGAA-3'' (Sense)<br>5'-CCGCTCGAGCTGCGCTGGAGAAGATGTCT-3'' (Antisense)
pGEX-6P-1-HOXA1 (1-1008)	5’-GGAGAATTCATGGACAATGCAAGAATGAA-3' (Sense)<br>5’-CCGCTCGAGTCAGTGGGAGGTAGTCAGAG-3' (Antisense)
pGEX-6P-1-HOXA1 (1-249)	5’-GGAGAATTCATGGACAATGCAAGAATGAA-3' (Sense)<br>5’-CCGCTCGAGGGAAGTCTGGTAGGTAGCCG-3' (Antisense)
pGEX-6P-1-HOXA1 (250-609)	5’-GGAGAATTCGGGAACCTGGGGGTGTCCTA-3' (Sense)<br>5’-CCGCTCGAGCTGCGCTGGAGAAGATGTCT-3' (Antisense)
pGEX-6P-1-HOXA1 (610-1008)	5’-GGAGAATTCACTTTTGACTGGATGAAAGT-3' (Sense)<br>5’-CCGCTCGAGTCAGTGGGAGGTAGTCAGAG-3' (Antisense)
pGEX-6P-1-HOXA1 (250-1008)	5’-GGAGAATTCGGGAACCTGGGGGTGTCCTA-3' (Sense)<br>5’-CCGCTCGAGTCAGTGGGAGGTAGTCAGAG-3' (Antisense)
pGEX-6P-1-HOXA1 (1-609)	5’-GGAGAATTCATGGACAATGCAAGAATGAA-3' (Sense)<br>5’-CCGCTCGAGCTGCGCTGGAGAAGATGTCT-3' (Antisense)
pBiFC-PITX2-VC155	5’-CCGAGATCTGGATGGAGACCAACTGCCGCAA-3' (Sense)<br>5’-CGGGGTACCCACGGGCCGGTCCACTGCAT-3' (Antisense)
pBiFC-HOXA1-VN173	5’-ATTTAAGCTTATGGACAATGCAAGAATGAA-3' (Sense)<br>5’-CGGGAATTCGGGTGGGAGGTAGTCAGAGTGT-3' (Antisense)
pEGFP-N1-PITX2	5’-CCCAAGCTTATGGAGACCAACTGCCGCAA-3' (Sense)<br>5’-CGCGGATCCCGCACGGGCCGGTCCACTGCAT-3' (Antisense)
pET28a-mCherry-HOXA1	5’-TGGACAGCAAATGGGTCGCGGAATGGACAATGCAAGAATGAA-3' (Sense)<br>5’-GCCCTTGCTCACCATGGATCCGTGGGAGGTAGTCAGAGTGT-3' (Antisense)
pET28a-EGFP-PITX2	5’-CCGGGATCCGAATGGAGACCAACTGCCGCAA-3' (Sense)<br>5’-CCCAAGCTTTCACGGGCCGGTCCACTGCAT-3' (Antisense)
pET28a-EGFP-PITX2 (ΔIDR)	5’-CCGGGATCCCAATGCGCGAAGAAATCGCTGTGTGGACC-3' (Sense)<br>5’-CCCAAGCTTTCTTGGCGGCCCAGTTGTTGTAGGA-3' (Antisense)
pEGFP-N1-PITX2 (ΔIDR)	5’-CCCAAGCTTATGCGCGAAGAAATCGCTGTGTGGACC-3' (Sense)<br>5’-CGCGGATCCCGCTTGGCGGCCCAGTTGTTGTAGGA-3' (Antisense)

PITX2, paired like homeodomain 2; HOXA1, homeobox A1.

Table S7 Oligonucleotide sets used for short hairpin RNAs and small interfering RNAs

Oligo Set	Sequences
sh-PITX2 #1	5’-CCGGTGCCGACTCCTCCGTATGTTTACTCGAGTAAACATACGGAGGAGTCGGCTTTTTG-3’ (Sense)
	5’-GATCCAAAAAGCCGACTCCTCCGTATGTTTACTCGAGTAAACATACGGAGGAGTCGGCA-3’ (Antisense)
sh-PITX2 #2	5’-CCGGTGCCGACTCCTCCGTATGTTTACTCGAGTAAACATACGGAGGAGTCGGCTTTTTG-3’ (Sense)
	5’-GATCCAAAAAAGGCCGAGCTATGCAAGAATGCTCGAGCATTCTTGCATAGCTCGGCCTA-3’ (Antisense)
sh-HOXA1 #1	5’-CCGGTCCCGCTGTTTACTCTGGAAATCTCGAGATTTCCAGAGTAAACAGCGGGTTTTTG-3’ (Sense)
	5’-GATCCAAAAACCCGCTGTTTACTCTGGAAATCTCGAGATTTCCAGAGTAAACAGCGGGA-3’ (Antisense)
sh-HOXA1 #2	5’-CCGGTCCCTCGGACCATAGGATTACACTCGAGTGTTAATCCTATGGTCCGAGGGTTTTTG-3’ (Sense)
	5’-GATCCAAAAACCCTCGGACCATAGGATTAACACTCGAGTGTAATCCTATGGTCCGAGGGA-3’ (Antisense)
sh-ATG5 #1	5’-CCGGTCCTTTCATTCAGAAGCTGTTTCTCGAGAAACAGCTTCTGAATGAAAGGTTTTTG-3’ (Sense)
	5’-GATCCAAAAACCTTTCATTCAGAAGCTGTTTCTCGAGAAACAGCTTCTGAATGAAAGGA-3’ (Antisense)
sh-ATG5 #2	5’-CCGGTGATTCATGGAATTGAGCCAATCTCGAGATTGGCTCAATTCCATGAATCTTTTTG-3’ (Sense)
	5’-GATCCAAAAA GATTCATGGAATTGAGCCAATCTCGAGATTGGCTCAATTCCATGAATCA-3’ (Antisense)
si-Scb	5’-GACGACCUGAUCGAAUUUACA-3’ (Sense)
	5’-UGUAAAUUCGAUCAGGUCGUC-3’ (Antisense)
si-RAB3A	5’-GGAUCAGAACUUCGACUACAU-3’ (Sense)
	5’-AUGUAGUCGAAGUUCUGAUCC-3’ (Antisense)

PITX2, paired like homeodomain 2; HOXA1, homeobox A1; ATG5, autophagy related 5; RAB3A, RAS-related protein Rab-3A.

Detailed Experimental Section

Cell lines: Gastric carcinoma cell lines NCI-N87 (CRL-5822), MKN-28 (JCRB0253), MKN-45 (JCRB0254), SUN-1 (CRL-5971), AGS (CRL-1739), and HGC-27 (CL-0107), cervical carcinoma HeLa (CCL-2), prostate cancer PC-3 (CRL-1435), and embryonic kidney HEK293T (CRL-11268) cells, were acquired from the American Type Culture Collection (ATCC, Manassas, VA) and Japanese Collection of Research Bioresources (JCRB, Tokyo, Japan). Cellular identity was verified through short tandem repeat (STR) genotyping, with experiments conducted within six months of thawing frozen stocks. Routine mycoplasma screening was performed using MycoAlert™ Mycoplasma Detection Kit (4460623, Thermo Fisher Scientific, Waltham, MA). Cells were propagated in a humidified incubator at 37°C and 5% CO_2, using RPMI 1640 medium prepared with 10% fetal bovine serum (Sigma, St. Louis, MO), and treated as specified with vacuolin-1 (673000, Sigma), ionomycin (407951, Sigma), ML-SI3 (MedChemExpress, Monmouth Junction, NJ), ML-SA1 (MedChemExpress), 1,6-hexanediol (88571, Sigma), or Nor (N-907, Sigma).

Real-time quantitative RT-PCR: RNA extraction was carried out using the PureLink RNA Mini Kit (12183018A, Thermo Fisher Scientific). The reverse transcription process was conducted with the QuantiTect Reverse Transcription Kit (205311, Qiagen, Hilden, Germany). For quantitative real-time PCR analysis, QuantiTect SYBR Green PCR Kit (204145, Qiagen) and specific primer pairs (Table S5) were utilized, with relative transcript quantification calculated according to the $2^{-\triangle\triangle Ct}$ method.

Western blotting: Cellular subfractionation was conducted utilizing a commercial Fractionation Kit (9038, Cell Signaling Technology, Danvers, MA). Total protein isolation from tissues or cultured cells employed RIPA Buffer (89900, Thermo Fisher Scientific). Immunoblotting analysis was subsequently performed, using specific primary antibodies specific for PITX2 (ab98297, Abcam Inc., Cambridge, MA), MCOLN1 (92176S, Cell Signaling Technology), RAB3A (ab302518), P16 (ab51243), P21 (ab109520), LC3 (ab192890), SQSTM1 (ab109012), ATG5 (ab108327), LAMP1 (ab24170), Na^+/K^+-ATPase (23565S), p-Akt^{308} (13038), p-Akt^{473} (4060), AKT (ab8805), E-cadherin (ab40772), N-cadherin (ab76011), Vimentin (ab92547), HOXA1 (ab230513), CTNNB1 (9566, Cell Signaling Technology), GST-tag (ab111947), MBP-tag (2396, Cell Signaling Technology Inc.), HA-tag (ab236632), FLAG-tag (ab205606), GAPDH (ab8245), or β-actin (ab6276). The original immunoblotting images were photographed directly by a ChemiDoc-IT 610 Imaging System (UVP, Upland, CA) under consistent exposure settings, with identical total exposure time and parameters for all samples. For visual clarity and presentation consistency, the entire images were equally subjected to minor adjustments of brightness and contrast using Adobe Photoshop 18.0 software (Adobe Systems Inc., San Jose, CA), which was performed to faithfully represent the original data.

Over-expression or silencing of genes: Human *PITX2* cDNA (816 bp) expression vector was acquired from Miaolingbio (Wuhan, China), while *HOXA1* cDNA (1008 bp) was syntheszed by TSINGKE Biotechnology (Beijing, China). Truncated variants of both genes were subsequently generated via polymerase chain reaction using primers specified in Table S6, and directionally cloned into either pCMV-HA or pCMV-3Tag-1A (Addgene, Cambridge, MA). For knockdown studies, oligonucleotides encoding shRNAs targeting *PITX2* or *HOXA1* (Table S7) were cloned into lentiviral delivery plasmid GV298 (GCD0316554, Genechem Co., Ltd., Shanghai, China). Stably transfected cancer cell populations were ultimately selected using puromycin (A1113803, Invitrogen, Carlsbad, CA).

Rescue of target gene expression: To counteract the changes in downstream gene expression resulting from *PITX2* depletion, *HOXA1* was reintroduced into established knockdown cell lines using its expression vector. In parallel, *HOXA1*-specific shRNAs (detailed in Table S7) were delivered into cancer cells employing

Neofect™ transfection reagent (Beijing, China) to reverse the effects on target gene expression elicited by *PITX2* over-expression.

Lentiviral packaging: HEK293T cells were co-transfected with lentiviral transfer plasmid and packaging constructs (psPAX2 and pMD2.G obtained from Addgene). After transfection, supernatants containing infectious viral particles were collected at 36 and 60 hours. The supernatants were then filtered using 0.45 μm PVDF filters (Sigma) and resuspended in phosphate-buffered saline (PBS).

Luciferase reporter assay: To evaluate human PITX2 activity, a luciferase reporter system was established by inserting oligonucleotides containing four canonical binding sites for PITX2 (Table S6) into pGL4.31 vector. This reporter construct was co-transfected into cancer cells with the *Renilla* luciferase reference vector pRL-SV40 (E2231, Promega, Madison, WI). The activity of firefly and *Renilla* luciferase was determined using GloMax 20/20 (Promega), enabling the calculation of normalized luciferase activity.

ChIP-qPCR: Cancer cells were seeded into 10-cm culture dishes and subjected to ChIP assay using the EZ-ChIP kit (MerkMillipore, Darmstadt, Germany). The assay was conducted with an antibody specific for PITX2 (ab221142). Real-time quantitative PCR (qPCR) was undertaken with QuantiTect SYBR Green PCR Kit (204145, Qiagen, Hilden, Germany) and primers (Table S5).

RNA-seq: Cancer cells (1×10^6) were lysed using RLT buffer (79216, Qiagen) combined with proteinase K digestion for RNA extraction with TRIzol® (15596018, Thermo Fisher Scientific). Library preparation and transcriptome sequencing were performed using the BGISEQ-500 platform (BGI Tech, Shenzhen, China). RNA-seq reads of 100-bp paired-end were aligned to genes using Kallisto v0.44.0, with transcript abundance expressed as fragments per kilobase of transcript per million mapped fragments (FPKM). The raw sequencing data were deposited in the GEO database under accession number GSE305469.

Immunofluorescence assay: Cells were cultured on glass coverslips, followed by incubation in 5% bovine serum albumin (BSA) solution for 1 hour. Subsequently, the cells were incubated with antibodies specific for PITX2 (ab98297, Abcam, 1:100 dilution) or HOXA1 (ab230513, Abcam, 1:100 dilution) overnight at 4°C. Afterward, the coverslips were incubated with corresponding secondary antibodies, including Alexa Fluor 488-conjugated goat anti-rabbit IgG (ab150077, Abcam, 1:1000 dilution) or Alexa Fluor 594-conjugated goat anti-rabbit IgG (ab150080, Abcam, 1:1000 dilution). The nuclei were counterstained with 4',6-diamidino-2-phenylindole (DAPI, 300 nmol/L) to visualize the cell nuclei. Fluorescence was observed using a fluorescence microscope.

BiFC assay: The BiFC assay was conducted according to established protocols. Human *HOXA1* (1008 bp) and *PITX2* (816 bp) cDNAs were respectively cloned into the pBiFC-VN173 or pBiFC-VC155 (Addgene, Table S6). Cancer cells were transfected with the recombinant plasmids. Twenty-four hours after transfection, the cells were fixed using 4% paraformaldehyde and subsequently stained using DAPI for 5 minutes. Fluorescence imaging was conducted via a confocal microscope (Nikon Instruments Inc.) with excitation and emission wavelengths set at 488 nm and 500 nm, respectively.

Co-IP and mass spectrometry: Co-IP was undertaken using 10 μg of antibodies for PITX2 (ab221142), HOXA1 (ab230513), GST-tag (ab111947), MBP-tag (2396, Cell Signaling Technology Inc.), FLAG-tag (ab205606), or HA-tag (ab236632, Abcam Inc.). Following elution from the magnetic bead complexes, proteins were quantified using western blot analysis. For proteomic analysis, proteins were enzymatically digested with trypsin, and the resulting peptides were extracted. The LC-MS/MS analysis was performed on a hybrid quadrupole-TOF LC/MS/MS system (SCIEX, Redwood City, CA). Each acquisition cycle included

a full-scan mass spectrum (m/z range: 350–1500, charge states: 2–5) followed by 40 MS/MS fragmentation events. The results were processed via ProteinPilot Software v5.0 and searched against the UniProt human protein database for sequence identification.

In vivo phase separation assay: Cancer cells expressing PITX2-pEGFP or endogenous expression were cultured on glass coverslips, and fixed with 4% paraformaldehyde for 10 minutes. Following two additional washes in PBS, cells were stained with Hoechst 33342 (Thermo Fisher Scientific) for 10 minutes. The cells were examined using a confocal microscope (Nikon A1R-SI) to analyze phase separation. The punctate structures were characterized by discrete clusters with a diameter greater than 0.5 μm.

In vitro phase separation assay: To prepare recombinant proteins, His-tagged *HOXA1* or *PITX2* constructs were transformed into *E. coli* BL21 cells (Thermo Fisher Scientific). The purification process was performed using HisTrap™ FF column (11-0004-59, Qiagen). For the phase separation assay, glass-bottom dishes were employed. Proteins were prepared at a concentration of 40 $\mu mol{\cdot}L^{-1}$ and incubated in droplet formation buffer (1 $mmol{\cdot}L^{-1}$ dithiothreitol, 10% glycerol, 50 $mmol{\cdot}L^{-1}$ Tris-HCl, pH 7.5), which also contained a crowding agent (10% PEG-8000). Observations were conducted using a Nikon A1R-SI confocal microscope with oil-immersion objectives.

FRAP: FRAP assays were performed on a confocal microscope with a 60× oil-immersion objective. *In vitro* studies incorporated photobleaching of droplets using 488-nm lasers at 50% power for 0.5 seconds. Sequential images were captured every three seconds post-bleaching for a total duration of 5 minutes. For *in vivo* studies, FRAP was performed on a confocal microscope at 37°C within a live-cell imaging chamber. Droplets were subjected to photobleaching using a 488-nm laser irradiation at 50% intensity for a duration of 0.5 seconds. Recovery kinetics were recorded over the designated timeframe, and recovery curves were analyzed via FIJI/ImageJ software.

SA-β-gal and EdU staining: To evaluate cellular senescence, cells were subjected to dual staining using the Senescence β-Galactosidase Staining Kit (Beyotime, C0602) and Click-iT™ EdU Detection Kit (Invitrogen). The protocol involved treating cells with EdU (diluted 1:1000 in medium) for 2 hours. Cells were treated with a 1× preservation buffer for a duration of 10 minutes, rinsed twice with PBS, and subsequently stained with β-galactosidase for 48 hours at 37°C. Finally, nuclei were counterstained with DAPI, and fluorescent images were captured using fluorescence microscopy.

Lysosome staining: Cells were cultured on glass-bottom dishes and treated with Lyso-Tracker Red (C1046, Beyotime, China) for 1 hour under standard culture conditions. After staining, cells were rinsed with PBS, counterstained with Hoechst 33342 for 15 minutes, and imaged using confocal microscopy.

Autophagic flux assay: Autophagic flux was assessed using GFP- and RFP-tagged LC3 constructs (Addgene). Cancer cells were cultured in 24-well plates and transfected with either GFP- or RFP-LC3 plasmids via Lipofectamine 3000 (Invitrogen). After 24 hours, the cells were fixed with 4% paraformaldehyde and visualized under a fluorescence microscope. Autophagosomes were identified as yellow puncta (GFP-RFP co-localization), while autolysosomes were detected as red puncta (RFP-only signals).

Transmission electron microscopy: Cancer cells were seeded onto 10-cm culture dishes and exposed to 2.5% glutaraldehyde for fixation. After 2–3 hours of fixation using 1% osmic acid, cancer cells were subjected to dehydration and subsequently embedded in paraffin. Ultra-thin sections with a thickness of 70 nm were prepared and sequentially stained with uranyl acetate and lead citrate. The lysosomal structures

were examined and imaged using transmission electron microscopy (JEM1230, JEOL, Tokyo, Japan).

Calcium imaging: To measure intracellular calcium levels, cancer cells were incubated with Fluo8-AM (5 $\mu mol \cdot L^{-1}$, ab142773, Abcam) under dark conditions. Following PBS washes, calcium fluorescence was analyzed using confocal microscopy.

ELISA: Protein concentrations in supernatant were assessed using ELISA kits targeting LGALS1 (Elabscience, E-EL-H1051c) and IGFBP7 (ab213790, Abcam Inc.). After centrifugation, the supernatant was stored at -80°C for subsequent analysis. The concentrations of cytokines (pg/ml) were standardized based on cell density per well (cells/ml), thereby calculating the cytokine production per cell.

Affinity purification: The FG beads (Nacalai Tesque, Inc., Kyoto, Japan) were incubated in a solution containing 20 $mmol \cdot L^{-1}$ Nor dissolved in N,N-dimethylformamide. Subsequently, 0.5 mg of Nor-immobilized beads were incubated with recombinant proteins for 2 hours at 4ºC. Following this, the beads were washed using 0.5% NP-40 lysis buffer. The eluted proteins were then subjected to western blot analysis for further characterization.

DSF assay: Recombinant GST-tagged HOXA1 or MBP-tagged PITX2 proteins were incubated in a solution of PBS containing SYPRO Orange dye (Invitrogen). Chemical compounds were then added to the reaction wells, and the temperature was raised from 40°C to 90°C at a rate of 1°C per minute. As the temperature increased, fluorescence intensity was continuously monitored at each 1°C increment and recorded as a function of temperature. The melting temperature (Tm) of the proteins was calculated using the Boltzmann equation.

Screening of FDA-approved drugs: Cancer cells were cultured at a density of 2×10^4 cells per well in 96-well plates under standard conditions, and exposed to compounds from the FDA Drug library (L2000-Z509781, Selleck) at a concentration of 10 $\mu mol \cdot L^{-1}$. To assess cellular viability, the MTT (MerkMillipore) colorimetric assay was employed.

Soft agar assay: Cancer cells (5×10^3 cells per well) were suspended in 0.05% Noble agar (A5431, Sigma) placed on 6-well plates containing 0.1% solidified Noble agar. The cells were incubated for 25 days under standard culture conditions. Then, the colonies were preserved via fixation and subsequently stained with a 0.5% crystal violet solution to enable visualization and enumeration under a microscope.

Cellular invasion assay: To assess cellular invasion, the assay utilized Matrigel matrix (354277, Corning, New York, NY). The procedure involved seeding starved cancer cells (1×10^5 cells per well) into the upper compartment of a Transwell system featuring an insertion module with 8.0-μm pore diameter (3428, Corning). Following a 24-hour incubation period, the cells were permitted to invade. Subsequently, the invaded cells were treated with 0.1% crystal violet for 10 minutes and subsequently counted via microscopy.

In vivo tumor formation and progression assays: All animal studies were performed in compliance with the guidelines for the care and use of laboratory animals established by the National Institutes of Health, and approved by the Experimental Animal Ethics Committee at Huazhong University of Science and Technology (Ethical Approval No. 2021-3229). Four-week-old BALB/c nude mice were randomly assigned to each group for *in vivo* tumor formation and progression studies. In the context of *in vivo* treatment studies, cancer cells stably transfected with red fluorescent protein (1×10^6 or 0.4×10^6 cells) were implanted into the dorsal flanks or tail veins of nude mice. The animals were randomly divided to receive either conditioned medium or Nor (50 mg/kg/day) via intravenous administration one week after the initial injection.

Subsequently, mice were monitored using the In-Vivo Xtreme II imaging system (Bruker Corporation, Billerica, MA) to assess fluorescence signals.

Human tissues: Human tissue samples were obtained with approval and oversight of the Ethics Review Board at Union Hospital, Tongji Medical College (Ethical Approval No. 2023-0604). The study was conducted in compliance with ethical principles set forth in the Declaration of Helsinki. At Union Hospital of Tongji Medical College, cancerous and adjacent normal specimens were collected during surgical procedures from patients diagnosed with gastric cancer. All participants provided written informed consent prior to their inclusion in the study, who had not undergone preoperative chemotherapy or radiation therapy. Fresh tumor specimens were collected intraoperatively, confirmed by pathological examination, and maintained at -80°C for further analysis.

Immunohistochemical staining: Immunohistochemistry and quantitative analyses were carried out, utilizing a monoclonal antibody targeting Ki-67 (ab15580; diluted at 1:100) or another specific for CD31 (ab28364; diluted at 1:50).

Statistical analysis: The data were analyzed by GraphPad 8.0 software (GraphPad Software, Boston, MA). All data are expressed as mean ± standard deviation (SD). Cutoff thresholds were established based on the median or average gene expression levels. Statistical comparisons between groups were conducted using two-tailed Student's *t*-test (for normally distributed parametric data) or one-way analysis of variance (ANOVA, for multiple distributed parametric data). The significance of overlaps was evaluated using Fisher's exact test. Kaplan-Meier curves were applied for analyzing survival data, while group comparisons were performed using two-sided log-rank test. All statistical analyses were two-tailed, with *P*-values less than 0.05 considered statistically significant.
